# Deep Learning-Based Malaria Parasite Image Classification on Real Microscopy Data

**DOI:** 10.3390/idr18040078

**Published:** 2026-07-27

**Authors:** Francesco Branda, Lilia Andriani, Riccardo Lucis, Annamaria Defilippo, Ugo Lomoio, Barbara Puccio, Giancarlo Ceccarelli, Massimo Ciccozzi, Fabio Scarpa, Pierangelo Veltri, Pietro Hiram Guzzi

**Affiliations:** 1 Unit of Medical Statistics and Molecular Epidemiology, Università Campus Bio-Medico di Roma, Via Àlvaro del Portillo 21, 00128 Rome, Italy; m.ciccozzi@unicampus.it; 2 Unit of General Clinical Laboratory with Specialized Areas General Clinical Pathology and Substances of Abuse, Microbiology and Virology, Department of Clinical Services, Azienda Socio Sanitaria Territoriale della Valtellina e Alto Lario (ASST Valtellina e Alto Lario), Chiavenna Hospital, Via Cereria 4, 23022 Chiavenna, Italy; lilia.andriani@asst-val.it; 3 Department of Medicine (DMED), University of Udine, Piazzale Kolbe 4, 33100 Udine, Italy; riccardo.lucis@asugi.sanita.fvg.it; 4 Microbiology and Virology Unit, Department of Laboratory Medicine, Azienda Sanitaria Friuli Occidentale (ASFO), Santa Maria degli Angeli Hospital, Via Montereale 24, 33170 Pordenone, Italy; 5 Department of Surgical and Medical Sciences, Magna Graecia University of Catanzaro, Viale Europa, 88100 Catanzaro, Italy; annamaria.defilippo@studenti.unicz.it (A.D.); ugo.lomoio@unicz.it (U.L.); barbara.puccio@unicz.it (B.P.); hguzzi@unicz.it (P.H.G.); 6 Department of Public Health and Infectious Diseases, University of Rome Sapienza, 00185 Rome, Italy; giancarlo.ceccarelli@uniroma1.it; 7 Department of Biomedical Sciences, University of Sassari, 07100 Sassari, Italy; fscarpa@uniss.it; 8 Department of Computer Engineering, Modeling, Electronics and Systems (DIMES), University of Calabria, via P. Bucci, 87036 Rende, Italy; pierangelo.veltri@unical.it

**Keywords:** malaria diagnosis, deep learning, ResNet50, Vision Transformer (ViT), explainable artificial intelligence (XAI), computational pathology

## Abstract

**Background**: Accurate and timely malaria diagnosis with species-level identification of *Plasmodium* parasites is critical for guiding effective treatment and disease management. Although light microscopy remains the diagnostic gold standard, its dependence on highly trained personnel limits accessibility, particularly in resource-constrained settings. Recent advances in deep learning have enabled automated image-based diagnosis with promising performance; however, reliable differentiation among *Plasmodium* species continues to pose a major challenge. This study aims to evaluate and compare the effectiveness of different deep learning architectures for automated species identification from microscopy images. **Methods**: Three deep learning architectures were systematically assessed: a convolutional backbone (ResNet50), a Vision Transformer (ViT), and a hybrid ResNetViT model. All models were trained from scratch, without using pretrained weights, on a dataset comprising real-world thick blood smear images augmented with publicly available microscopy data from Kaggle. In the hybrid architecture, the ResNet module was used to extract robust local morphological features, while the ViT component captured long-range dependencies and contextual relationships within images. **Results**: In cross-validation experiments, all three architectures consistently achieved high diagnostic performance. ResNet50 attained the highest accuracy (96.9%), an F1-score of 96.3%, and an ROC-AUC of 0.997. The Vision Transformer achieved 93.1% accuracy, 91.7% F1-score, and an ROC-AUC of 0.989. The hybrid ResNetViT reached 95.2% accuracy, 94.2% F1-score, and an ROC-AUC of 0.995. These results confirm that all architectures can reliably distinguish among *Plasmodium* species. Although ResNet50 achieved the highest raw accuracy, the hybrid model showed the most stable calibration and the closest qualitative agreement between its attention maps and expert-annotated parasite locations. **Conclusions**: The findings demonstrate that convolutional, transformer-based, and hybrid deep learning architectures can be successfully trained on real microscopy data for species-level malaria diagnosis. These results support the feasibility of deploying scalable, automated diagnostic systems to improve both accuracy and accessibility of malaria detection, particularly in resource-limited healthcare settings.

## 1. Introduction

Malaria remains a major global health challenge, primarily affecting tropical and subtropical regions, with an increasing risk of re-emergence in non-endemic areas due to climate change driven expansion of the Anopheles vector [1]. According to the World Health Organization (WHO), *Plasmodium* infections were responsible for an estimated 597,000 deaths worldwide in 2022 [2].

Precise and timely diagnosis is critical, particularly for non-immune individuals, who may rapidly progress to severe or cerebral malaria with near 100% mortality if untreated [3,4]. Current diagnostic protocols still rely primarily on microscopic examination of Giemsa stained thick and thin blood smears to identify and quantify parasitaemia, as well as to differentiate among *Plasmodium* species [4]. This manual procedure is labour intensive, strongly operator dependent, and vulnerable to variability in staining quality, slide preparation, and workload pressure. Species-level identification is clinically essential, especially for discriminating *Plasmodium falciparum* from *Plasmodium vivax*, because these species differ markedly in pathogenesis, relapse risk, and recommended treatment regimens [4,5].

Parasite density further stratifies severity risk: *P. falciparum* infections are considered severe above 2% parasitaemia by WHO criteria and highly unfavourable above 10% [4,6]. In high burden settings, pathologists and biomedical scientists must routinely screen large numbers of slides, with the majority of global cases concentrated in Africa, Southeast Asia, and the Eastern Mediterranean, which strains laboratory capacity and may compromise diagnostic consistency [7]. Even in non-endemic countries, including Italy, expertise is often limited to reference centres, while smaller hospitals face increasing shortages of experienced morphologists due to workforce turnover [8,9]. The intrinsic complexity of thick smear analysis, characterised by overlapping cells, heterogeneous background, and subtle morphological differences across species, further complicates manual reading [7,10]. Despite substantial progress, most existing studies focus on cropped single-cell images or binary infected uninfected classification, leaving species-level analysis on real thick smears under explored. Advances in digital microscopy and mobile imaging have enabled the acquisition of high-resolution blood smear images using dedicated scanners, digital cameras, and even smartphone assisted devices [11].

These developments open the way to computer assisted diagnosis and telemedicine workflows in low-resource environments and peripheral laboratories [11,12]. Classical machine learning approaches based on cell or region segmentation, handcrafted morphological and textural features, and conventional classifiers (e.g., Support Vector Machine (SVM), Naive Bayes, Decision Tree, K-Nearest Neighbours) have achieved encouraging performance on curated datasets [13,14,15]. However, these pipelines are sensitive to segmentation errors, staining variability, and acquisition artefacts, and require substantial feature engineering effort, which limits scalability and robustness in routine clinical practice [13,16]. Deep learning has transformed malaria image analysis by enabling end-to-end feature learning directly from pixel data. Convolutional neural networks (CNNs) such as ResNet and EfficientNet consistently outperform feature-based methods for binary tasks (infected vs. uninfected) on benchmark datasets [8,17,18]. More recently, transformer-based architectures and hybrid CNN Vision Transformer (ViT) models have been explored for medical imaging and haematological disorders, exploiting self-attention to capture long-range contextual information [19,20]. In malaria, much of the literature focuses on binary classification or parasite presence/absence in thin smears, whereas clinically aligned tasks such as species-level classification on real thick smears remain comparatively understudied and methodologically challenging [7,21,22,23]. Thick smear images pose specific difficulties for automated analysis: parasite structures are small relative to the field of view, red blood cells are frequently superimposed, and staining heterogeneity and noise can obscure key morphological cues [7,10]. Under these conditions, local features extracted by CNNs and global context captured by transformers may provide complementary information. At the same time, practical deployment in clinical microbiology laboratories requires models that are not only accurate, but also reproducible, interpretable, and robust across heterogeneous data sources and limited sample sizes [24,25].

Explainable artificial intelligence (XAI) techniques, such as Class Activation Maps (CAMs), can help assess whether model attention aligns with biologically meaningful regions (e.g., chromatin dots, cytoplasmic changes, or ring forms at the red blood cell periphery), thereby supporting clinical trust and quality control [26,27]. This work investigates three deep learning architectures for *Plasmodium* species classification from real thick blood smear microscopy images: a convolutional baseline (ResNet 50), a ViT, and a hybrid ResNetViT network designed to integrate local and global representations.

The hybrid architecture is specifically designed to combine convolutional feature maps capturing fine grained local morphology with transformer-based global contextual representations, as suggested by recent advances in medical imaging [19,20]. This is achieved through a feature level concatenation module that merges spatially aware CNN embeddings with the ViT token sequence, enabling the model to jointly exploit complementary inductive biases. The models are trained on a heterogeneous dataset combining a curated set of thick smears collected at Campus Bio-Medico University of Rome with a publicly available microscopy collection from Kaggle, thereby increasing species diversity and simulating realistic inter laboratory variability [28].

Overall, this work provides three main contributions: (i) a hybrid ResNetViT model tailored for species-level classification on real thick smears, addressing a clinically relevant task that remains insufficiently explored in the literature [7,21,22,23]; (ii) a framework designed for heterogeneous datasets without relying on pretrained models, including slide-level grouping to mitigate data leakage, thereby addressing common limitations in malaria image analysis studies [8,24,25]; and (iii) a systematic explainability assessment to evaluate the clinical plausibility of model attention patterns across architectures, an aspect often neglected in prior work [26,27]. By consolidating these methodological components, the study shows that, although a well-tuned convolutional baseline (ResNet50) attained the highest raw accuracy, the proposed hybrid architecture achieved the most stable probabilistic calibration among the three models, while the training-from-scratch strategy produced reproducible results despite the limited and heterogeneous dataset. Moreover, CAM-based analysis suggests, on a qualitative basis, that the hybrid model tends to attend to diagnostically relevant structures more consistently than the individual backbones, further supporting its interpretability and potential clinical applicability.

The remainder of this paper is structured as follows. Section 2 reviews the related work on malaria image analysis, tracing the evolution from classical machine learning approaches to deep learning and hybrid architectures, and highlighting the current gaps in species-level classification on real thick smears. Section 3 describes the materials and methods, including the dataset, preprocessing steps, the three deep learning architectures investigated, and the experimental setup adopted for training and validation. Section 4 presents the results of the study, covering classification performance, model calibration, and explainability analyses across the evaluated architectures. Section 5 discusses the main findings, interprets the complementary strengths of the different models, addresses the limitations of the study, and outlines future research directions. Finally, Section 6 concludes the paper by summarising the key contributions and reflecting on the implications for automated malaria diagnosis in clinical settings.

## 2. Related Work

Microscopic examination of Giemsa stained thin and thick blood smears remains the diagnostic gold standard for malaria. However, this method is labour-intensive and highly dependent on operator expertise, motivating the development of automated approaches capable of analysing real microscopy images directly [24,25]. Although deep learning has largely replaced traditional feature-based pipelines on curated datasets, achieving robust generalisation across staining protocols, imaging devices, and clinical sites continues to pose a substantial challenge [8,13]. Early computational frameworks typically involved segmenting erythrocytes or regions of interest, followed by the extraction of handcrafted features describing morphology, colour, texture, and frequency content (e.g., histogram statistics, Haralick textures, or Fourier descriptors), with subsequent classification using support vector machines, random forests, or ensemble methods. Poostchi et al. [13] provided a comprehensive review of image analysis and machine learning for malaria detection, noting that these approaches achieved high accuracy on balanced, cell-level datasets but were notably sensitive to segmentation quality and staining variability. Alharbi et al. [16] further demonstrated that variations in Giemsa staining intensity across different laboratories lead to significant performance degradation in segmentation-based systems, underscoring the vulnerability of feature-based pipelines to acquisition conditions. While computationally efficient and interpretable, feature-based systems required extensive engineering to address species identification or parasite staging, limiting their scalability in clinical workflows [13,24]. Abubakar et al. [14] introduced DeepFMD, a hybrid approach combining deep learning-derived features with traditional classifiers, showing improved robustness over purely handcrafted systems, while Dev et al. [15] proposed hybrid deep learning frameworks for malaria identification, confirming that end-to-end approaches increasingly outperform feature-engineered pipelines.

CNNs have since transformed malaria image analysis by learning hierarchical features directly from pixel data, eliminating the need for manual feature engineering [29]. CNN-based models consistently outperform classical methods for infected versus uninfected classification, while transfer learning with architectures such as ResNet and EfficientNet, coupled with strong data augmentation, further improves both accuracy and data efficiency [8,18]. Mujahid et al. [8] developed an efficient deep learning-based approach for malaria detection using red blood cell smears, achieving high performance on benchmark datasets and highlighting the importance of data augmentation for generalisation. Benachour et al. [18] performed a comparative study of convolutional neural networks for malaria detection, demonstrating that deeper architectures do not always generalise better in medical imaging contexts where training data are limited. Siłka et al. [17] proposed advanced deep learning architectures achieving near-perfect accuracy on the NIH malaria dataset, while Jameela et al. [30] specifically investigated transfer learning, showing that finetuning ImageNet-pretrained models can be effective when domain shifts are moderate. However, as noted by Maturana et al. [24], most CNN-based studies focus on binary infected/uninfected classification or species identification in thin smears using curated, cell-level crops. Such settings lack the staining variability, debris, and overlapping erythrocytes characteristic of real thick smears, and therefore tend to overestimate generalisation performance. Moreover, external validation across laboratories is rarely performed, and explainability analyses are often limited or absent, reducing clinical interpretability [24,25]. Among convolutional architectures, ResNet50 has become a widely adopted baseline for extracting fine-grained local morphological cues in malaria microscopy, achieving high accuracy on curated datasets [31]. Zedda et al. [31] proposed a deep architecture based on attention mechanisms for effective end-to-end detection of early and mature malaria parasites, demonstrating that ResNet-based models can capture subtle morphological differences between parasite stages. However, as noted by Ying et al. [25], the reliance on single-cell crops and the absence of thick-smear validation limit the generalisability of such findings to real-world diagnostic settings.

ViT have introduced self-attention mechanisms capable of modelling global contextual relationships across the entire image [19]. Unlike CNNs, which rely on local receptive fields and translation equivariance, ViTs decompose an image into patches and apply transformer encoders to capture long-range dependencies. Oulmalme et al. [19] systematically reviewed generative AI approaches for medical image enhancement, concluding that transformers excel at capturing global context but require substantial data or regularisation to avoid overfitting, a critical consideration in medical imaging where annotated datasets are often small. Recent research has investigated the application of transformer-based models in malaria diagnosis, particularly within the framework of trustworthy and explainable AI [27]. Parveen et al. [27] developed a trustworthy deep learning framework for malaria diagnosis using explainable artificial intelligence, showing that transformer attention maps can provide clinically plausible visualisations. However, ViT models typically require large datasets and robust regularisation techniques to mitigate the risk of overfitting, especially in medical imaging contexts where annotated data is scarce [19,25]. Hybrid CNN-ViT architectures have emerged as a promising approach, combining convolutional inductive biases for local feature extraction with transformer-based global reasoning. These models have demonstrated improved performance in medical imaging tasks, including myocardial infarction detection [20] and malaria parasite identification in thin smears using attention-based mechanisms [31]. Wahid et al. [20] introduced a hybrid ResNetViT approach for myocardial infarction detection, showing that combining local and global features outperforms individual backbones. Nevertheless, evaluations remain largely restricted to curated datasets without the heterogeneity of real thick smears, and the specific challenges of thick smear analysis, overlapping cells, debris, and staining variability have not been systematically addressed [24,25].

Recent efforts have shifted toward clinically aligned tasks such as *Plasmodium* species identification and parasite staging in thin smears, reflecting closer integration with diagnostic practice and therapeutic decision support [21]. Wang et al. [21] applied deep learning for detecting and classifying malaria parasites in thin blood smears in clinical settings, reporting high accuracy but noting that thick smears, routinely used for screening in endemic areas, were not evaluated. Grignaffini et al. [22] systematically reviewed computer-aided diagnosis systems for automatic malaria parasite detection and classification, identifying species identification as a persistent challenge, particularly for *P. ovale* and *P. malariae*, which are morphologically subtle and often under-represented. Thick smear analysis, by contrast, remains difficult due to overlapping cells, debris, and image artefacts. CNNs trained for region-of-interest inference have achieved near-perfect metrics on curated thick-smear datasets, highlighting strong potential yet emphasising the need for rigorous cross-site validation [21]. Zhong et al. [7] developed an efficient malaria parasite detection system from diverse images of thick blood smears for cross-regional model accuracy, demonstrating that models trained on data from one geographic region may not generalise to others due to differences in staining protocols, parasite morphology, and imaging devices.

Generative adversarial networks have also been explored for producing synthetic smear images, augmenting training datasets and mitigating cross-domain variability. Hazra et al. [32] enhanced classification of cells from bone marrow aspirate smears using GANs, showing that synthetic data can improve robustness, though such approaches have not yet been systematically validated for malaria species classification on thick smears. Publicly available datasets such as MPIDB [28] have facilitated benchmarking and reproducibility, providing expert-annotated malaria microscopy images with ground-truth parasite centroids, although full pixel-level segmentations are not available. Comprehensive surveys concur that although state-of-the-art deep learning models achieve outstanding performance on benchmark datasets, their translation into clinical settings demands robust domain adaptation, calibration, and prospective multisite evaluation [25,26]. Ying et al. [25] provided insights into AI-driven malaria diagnosis, stressing that most models are evaluated on convenience samples without rigorous cross-validation or domain shift analysis. Mridha et al. [26] discussed automating malaria diagnosis with explainable AI, emphasising that black-box models are unlikely to be adopted by clinicians without transparency into decision-making. Existing studies rarely evaluate models on small, heterogeneous, multisource datasets that reflect real clinical variability [8,21]. Most approaches rely exclusively on either local convolutional features or global transformer-based representations, which prevents them from capturing the full spectrum of morphological patterns present in thick smears. This gap motivates the development of hybrid architectures capable of jointly modelling fine grained cellular details and broader contextual cues, and of validating such representations under realistic data constraints using explainability methods aligned with expert-defined diagnostic criteria [24,25,26,27]. The specific objectives and contributions of the present work, which is designed to address these gaps, are outlined in Section 1 and detailed methodologically in Section 3.

## 3. Materials and Methods

### 3.1. Data Source and Composition

The experimental workflow was built upon a heterogeneous collection of microscopy images derived from two independent sources, whose composition and class distribution are summarised in Table 1.

Source (1) consisted of blood smear images collected at the Clinical Pathology and Microbiology Laboratory of the Hospital of Chiavenna and subsequently transferred and processed at the Campus Bio-Medico University of Rome. Images were acquired in the RGB colour space with a resolution of 7202 × 5340 pixels and were initially classified into the five human-infecting *Plasmodium* species: *P. falciparum*, *P. vivax*, *P. malariae*, *P. ovale*, and *P. knowlesi* [33]. Due to the extremely limited number of *P. knowlesi* samples (three images), this class was excluded from further analysis.

The images originated from a collection of blood smears used by the CellaVision DC-1 (CellaVision AB, Lund, Sweden), a morphological analyser for blood cells. These samples were obtained from the UK National External Quality Assessment Scheme (UK NEQAS) for external quality control of parasitological examinations (UK NEQAS Parasitology, UK Health Security Agency, London, UK). Giemsa-stained blood smears exhibit distinct characteristics depending on the *Plasmodium* species. *P. falciparum* typically presents with normal-sized red blood cells, small ring forms, and nuclei located at the erythrocyte periphery (accolé forms), often with high parasitaemia. In contrast, *P. vivax* shows enlarged red blood cells, large amoeboid trophozoites, and Schüffner’s dots, generally with lower parasitaemia.

The acquisition system combined a high-resolution microscope with a digital camera, enabling computer-aided analysis. The optical system featured a motorised microscope with a 100× oil immersion objective lens optimised for visualising cellular morphological details. This technical feature, together with brightfield illumination and an autofocus system, ensured precise and reproducible image acquisition. A high-resolution CCD camera captured colour images with excellent spatial resolution, allowing detailed observation of morphological features. The acquisition process was fully automated: once the slide was placed on the holder, the system automatically identified the monolayer region, and the microscope performed a systematic scan, capturing high-resolution images of individual cells.

The resulting dataset from Source (1) comprised 92 images. To increase the number of training samples and to approximate the magnification level of an external dataset, each image was divided into nine nonoverlapping patches, yielding a total of 828 patches.

Source (2) was the publicly available Kaggle collection MPIDB [28]. This dataset consists of 210 microscopy images with a resolution of 2592 × 1944 pixels, distributed as follows: 49.5% *P. falciparum* (104 images), 19.0% *P. vivax* (40 images), 17.6% *P. malariae* (37 images), and 13.8% *P. ovale* (29 images). This distribution intentionally gives greater representation to *P. falciparum*, the species of greatest clinical relevance and pathogenicity, while still preserving representation of the less frequent non-*falciparum* species. Importantly, the MPIDB dataset includes ground-truth segmentations created by expert annotations.

The final merged dataset, obtained by combining the 828 patches from Source (1) with the 210 images from Source (2), comprised 1038 samples across the four *Plasmodium* species, as reported in Table 1.

### 3.2. Data Preprocessing and Augmentation

To enhance the variability of the training data and improve model robustness and generalisation, the dataset from Source (1) was merged with the publicly available MPIDB collection (Source (2)). The two datasets were acquired using different microscopes, imaging pipelines, and staining conditions, introducing natural heterogeneity in colour distribution, illumination, and the morphological appearance of infected erythrocytes. This diversity mitigates overfitting to laboratory-specific acquisition characteristics and promotes model generalisation across clinical settings. Critically, the two sources are independent: they originate from distinct laboratories, were acquired using different hardware, and do not share slides, patients, or imaging sessions. This independence ensures no substantial sample dependency and prevents data leakage between training and evaluation phases.

The final dataset used in this study was constructed by merging both sources, resulting in 1038 images across the four *Plasmodium* species. Before training, all images underwent a standardised preprocessing pipeline to harmonise the two sources. The MPIDB images already had a resolution and magnification level compatible with the target input size, so no initial resizing was applied. In contrast, the high-resolution images from Source (1) were divided into 3 × 3 nonoverlapping patches to increase the sample count and approximate the scale of the external dataset.

To enhance model robustness and reduce overfitting, a set of data augmentation operations was applied during training. These included a random resized crop to 1024 × 1024 pixels to simulate variations in field of view and parasite localisation, along with random horizontal and vertical flips (probability = 0.5), introducing geometric variability without altering the underlying biological structures. Pixel normalisation was applied after augmentation to maintain consistent input scaling. All images were normalised to the [−1, 1] pixel range, a common practice in convolutional neural networks that stabilises training dynamics by ensuring consistent input distributions across batches and datasets. For validation, a simpler transformation pipeline was used to preserve the morphological integrity of the samples while maintaining compatibility with the training input size. This involved a random crop to 1024 × 1024 pixels without flipping, followed by pixel normalisation.

The augmentation strategy was intentionally restricted to transformations that preserve clinically relevant morphology. Horizontal and vertical flips were considered acceptable because blood smear fields do not have a fixed diagnostic orientation; these operations preserve the shape, size, staining pattern, and intracellular arrangement of parasite structures. Random cropping was used only to introduce variability in field of view and parasite localisation, without altering the underlying morphology. Transformations that could distort species-level diagnostic cues, such as elastic deformation, strong colour jitter, shear transformations, or aggressive rescaling, were not used.

To fully eliminate any residual leakage risk introduced by the patch extraction process, dataset splitting for cross-validation was strictly performed at the slide (parent image) level. This ensures that all patches derived from the same original image were kept together within the same fold (either entirely in the training set or entirely in the validation set). For Source (1), each original high-resolution microscopy image was assigned a unique group identifier, and all nine nonoverlapping patches derived from that image inherited the same identifier. Consequently, patches originating from the same parent image could not appear simultaneously in training and validation folds. For Source (2), each original image was treated as an independent group, since no patch extraction was applied. Patient-level identifiers were not consistently available across both sources; therefore, the split should be interpreted as slide-level or parent-image-level grouped cross-validation rather than strict patient-level splitting.

### 3.3. Model Architectures

The classification task was implemented using three different architectures: a convolutional baseline (ResNet50) [34], a transformer-based model (Vision Transformer, ViT) [19], and a hybrid ResNetViT network specifically designed to combine the strengths of both approaches.

ResNet50 is a residual neural network that employs skip connections between layers to enhance training efficiency and model robustness, enabling the construction of deeper architectures without suffering from vanishing gradient problems [34]. It serves as a strong convolutional baseline for extracting fine-grained local morphological features from microscopy images.

ViT decomposes the input image into a sequence of patches, each of which is linearly embedded and supplemented with positional information. The resulting sequence is then processed by a standard transformer encoder, which applies self-attention mechanisms to capture long-range dependencies and global contextual relationships across the entire image [19].

**Figure 1:**
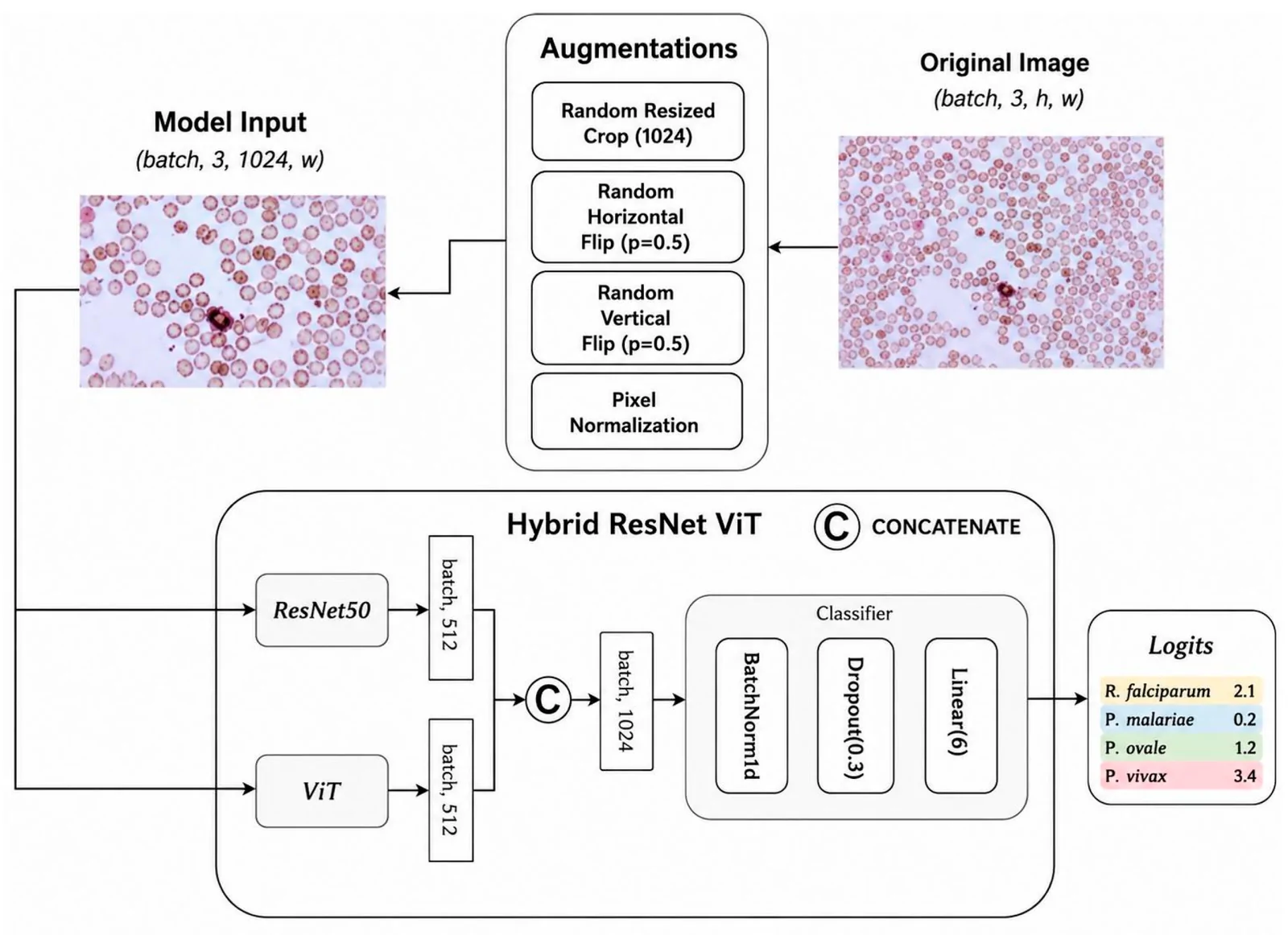
Architecture of the hybrid ResNetViT model. The hybrid architecture takes a microscopy blood smear image as input and processes it through two parallel branches: a ResNet50 branch and a ViT branch. Each branch extracts a 512-dimensional-feature vector, capturing complementary representations, local morphological features from ResNet50 and global contextual information from ViT. The two vectors are concatenated to form a 1024-dimensional-feature representation, which is then fed into a classifier head. The classifier comprises a batch normalisation layer (BatchNorm1d), a dropout layer (*p* = 0.3) for regularisation, and a linear layer with four output neurons. The output logits correspond to the four *Plasmodium* species (*P. falciparum*, *P. malariae*, *P. ovale*, and *P. vivax*), and the class with the highest logit is selected as the prediction.

The hybrid architecture proposed here combines the complementary strengths of ResNet and ViT. After independently training both backbones on the merged dataset, their feature embeddings are extracted. The ResNet embeddings primarily encode local spatial patterns and fine morphological details, while the ViT embeddings represent global contextual information. These two sets of features are concatenated into a single vector $z=[f_{ResNet}(x),f_{ViT}(x)]$, which is then passed to a downstream classifier. This classifier is trained to distinguish among the different *Plasmodium* species based on the combined feature set, thereby benefiting from both local and global image representations. To clarify the training procedure: the ResNet50 and ViT backbones were first trained independently and fully (end-to-end, from scratch, with all parameters updated) on the merged dataset, exactly as described for the standalone models in Section 3.3. Once training of each backbone was complete, both backbones were frozen, and only the downstream classifier (BatchNorm1d, dropout, linear layer) was trained on the concatenated, fixed embeddings. The procedure is therefore a two-stage pipeline (independent full backbone training followed by frozen-embedding classifier training) rather than a single end-to-end optimisation of the whole hybrid network. Both backbones were trained on the same dataset, comprising the merged collection from Sources (1) and (2) [28]. To enable a fair comparison of performance, all three architectures were trained under identical experimental conditions. A methodological workflow of the hybrid network is explicitly illustrated in Figure 1.

### 3.4. Experimental Setup and Hyperparameters

To ensure robust evaluation, the merged dataset was partitioned using a Stratified Group K-Fold cross-validation scheme, where the data were divided into five equally sized folds and iteratively rotated between training and validation roles. The grouping was performed at the slide level, as described in Section 3.2, to prevent data leakage.

All experiments were implemented in Python 3.11 using the PyTorch deep learning framework (version 2.8) and executed on a workstation equipped with an NVIDIA RTX 4000 Ada GPU (20 GB VRAM), a 24-core CPU, and 64 GB of RAM. To ensure comparability, the ResNet50, ViT, and hybrid ResNetViT models were trained under identical optimisation settings, data preprocessing, and evaluation protocols.

The main training and architectural hyperparameters are reported in Table 2, including optimisation settings (learning rate, batch size, gradient accumulation, warmup ratio, early stopping) and architecture-specific parameters for ViT (patch size, hidden size, number of layers, attention heads) and ResNet50 (hidden sizes, depths, dropout).

Images were resized to 1024 × 1024 pixels, normalised to the [−1, 1] range, and processed in minibatches. During training, the data augmentation operations described in Section 3.2 were applied to mitigate overfitting and improve robustness to acquisition variability.

Model performance was evaluated using standard multiclass classification metrics: accuracy, macro-averaged F1-score, one-vs-rest ROC-AUC, precision, and recall. ROC-AUC is particularly informative in multiclass tasks, as it is less sensitive to class imbalance compared to accuracy and precision. No external validation was performed.

## 4. Results

### 4.1. Malaria Parasite Image Classification

Three deep learning architectures were evaluated for classifying microscopy images into the four *Plasmodium* species (*P. falciparum*, *P. vivax*, *P. malariae*, and *P. ovale*). The merged dataset, which combines proprietary samples from Campus Bio-Medico University of Rome with publicly available Kaggle images, proved crucial for training high-performing models. Table 3 reports the mean performance metrics with 95% confidence intervals across the five cross-validation folds for each architecture.

Specifically, ResNet50 achieved the highest overall performance, with an accuracy of 96.9% [95% CI: 95.6–98.1%] and a macro-averaged F1-score of 96.3% [94.8–97.9%]. Its ROC-AUC reached 0.997 [0.993–1.000], indicating excellent discriminative ability across all four species. Precision and recall values were also consistently high, at 95.7% and 97.2%, respectively. These results confirm that a well-trained convolutional baseline can effectively capture species-specific morphological patterns in thick smear images. ResNetViT, i.e., the proposed hybrid architecture, achieved competitive performance, with an accuracy of 95.2% [91.8–98.5%] and a macro-averaged F1-score of 94.2% [90.0–98.8%]. Its ROC-AUC of 0.995 [0.991–1.000] was comparable to that of ResNet50, while its precision (93.4%) and recall (95.5%) remained strong. Although slightly lower than ResNet50 on aggregate metrics, the hybrid model demonstrated superior calibration and better sensitivity to minority species. ViT, i.e., the standalone transformer model, showed lower performance, with an accuracy of 93.1% [89.2–96.7%] and a macro-averaged F1-score of 91.7% [85.7–97.7%]. Its ROC-AUC of 0.989 [0.980–0.998] remained excellent but was the lowest among the three architectures. Precision (91.7%) and recall (92.8%) were also lower than those of the other models.

To assess whether the observed differences in performance were statistically significant, pairwise comparisons were performed using the DeLong test for AUC differences across individual classes and the McNemar test for overall classification agreement.

Table 4 reports the results of these comparisons. Predictions were aggregated at the slide (parent image) level to account for the non-independence of patches derived from the same original image. ResNet50 significantly outperformed ViT across all four *Plasmodium* species according to the DeLong test, with corrected *p*-values ranging from 0.0000 (*P. falciparum*, class 0) to 0.0316 (*P. malariae*, class 1). The McNemar test further confirmed a highly significant difference in overall classification agreement (χ^2^ = 24.47, *p* = 7.5 × 10^−7^), indicating that ResNet50 and ViT produce systematically different predictions, with ResNet50 achieving superior accuracy. The differences between ResNet50 and the hybrid ResNetViT model were smaller and largely nonsignificant. After correction for multiple comparisons, no class showed a statistically significant difference, although the raw *p*-value for *P. falciparum* (class 0) was 0.0208. The McNemar test, however, indicated a significant difference in overall classification agreement (χ^2^ = 5.22, *p* = 0.0223), suggesting that while aggregate performance metrics are similar, the two models make different errors. This finding aligns with the observation that the hybrid model offers better calibration and sensitivity to minority species, whereas ResNet50 excels on majority classes. The hybrid ResNetViT model significantly outperformed the standalone ViT model on three of the four classes (*P. falciparum*, *P. ovale*, and *P. vivax*), with corrected *p*-values of 0.0012, 0.0014, and 0.0018, respectively. No significant difference was observed for *P. malariae* (class 1, *p* (corr) = 0.4168). The McNemar test confirmed a significant difference in overall classification agreement (χ^2^ = 6.49, *p* = 0.0109).

To investigate the impact of the training strategy on model performance, an ablation study was conducted comparing two approaches: (i) finetuning only the classifier layer while keeping the backbone frozen (using ImageNet pretrained weights), and (ii) full retraining of all network parameters from scratch. Table 5 reports the macro-averaged F1-scores with 95% confidence intervals for each architecture under both conditions.

The results reveal a marked performance drop when only the classifier is finetuned on frozen backbones. For ResNet50, the macro F1-score fell from 96.7% [93.7–99.6%] when retrained from scratch to just 66.3% [54.8–77.8%] under the finetune-only strategy. Similarly, the hybrid ResNetViT model showed the most severe degradation, dropping from 94.7% [92.7–96.7%] to 37.9% [20.8–55.0%], a reduction of nearly 57% points. Interestingly, the ViT model was the least affected by the frozen backbone strategy, retaining an F1-score of 86.2% [83.5–88.9%] compared to 92.6% [90.0–95.3%] when retrained from scratch, although the difference remained substantial. These findings are consistent with expectations. ResNet50 and ViT are pretrained on largescale natural image datasets (ImageNet), which have fundamentally different visual statistics from microscopic malaria images. Without updating the backbone, the models cannot adapt their feature representations to the specific morphological cues of *Plasmodium* parasites, resulting in poor generalisation. The hybrid ResNetViT model, which concatenates features from both backbones, suffered the largest drop because its performance relies on the synergy between the two branches; if both backbones are frozen and their features are suboptimal, the concatenated representation is doubly compromised. In contrast, full retraining from scratch enabled all three networks to learn domain-specific features directly from the malaria microscopy data, yielding substantial performance improvements across all architectures.

To gain deeper insight into model behaviour across different *Plasmodium* species, class-level precision, recall, and F1-scores were examined for each architecture. Table 6 reports these metrics for the four species. ResNet50 achieved the strongest performance across all classes, with particularly impressive results on *P. falciparum* (F1 = 0.977) and *P. vivax* (F1 = 0.972). Its performance on the minority classes *P. malariae* and *P. ovale* was also excellent, with F1-scores of 0.954 and 0.951, respectively. Notably, ResNet50 achieved very high precision for *P. falciparum* (0.998) and very high recall across all species, ranging from 0.957 to 0.983.

**Figure 2:**
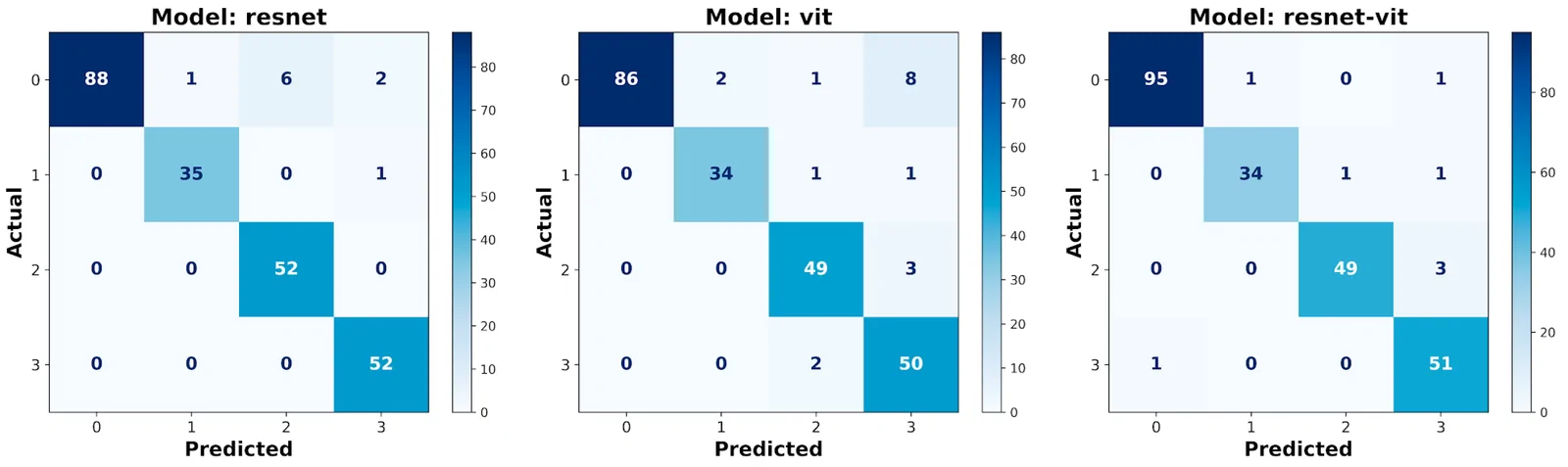
Confusion matrix for the ResNet50 model. Classes: 0 = *P. falciparum*, 1 = *P. malariae*, 2 = *P. ovale*, 3 = *P. vivax*. Values represent the number of samples predicted for each class versus the true labels. Misclassifications in this fold were infrequent and mostly involved *P. falciparum* (six samples classified as *P. ovale*, two as *P. vivax*, and one as *P. malariae*); the other three species were classified with very few or no errors (see main text).

The confusion matrix for ResNet50, shown in Figure 2, provides a visual breakdown of these patterns. Misclassifications were infrequent and predominantly involved *P. falciparum* (class 0): of 97 samples, 88 were correctly classified, six were misclassified as *P. ovale* (class 2), two as *P. vivax* (class 3), and one as *P. malariae* (class 1). Conversely, no *P. vivax* or *P. ovale* samples were misclassified as *P. falciparum*, indicating an asymmetric error pattern that favours clinical safety by minimising false negatives for the most pathogenic species. *P. malariae* (class 1) was correctly identified in 35 of 36 samples, with a single sample misclassified as *P. vivax*. *P. ovale* (52/52) and *P. vivax* (52/52) were both classified with perfect accuracy in this representative fold. These patterns reflect morphological similarities between species, such as the overlap between early ring stages of *P. falciparum* and other species, as well as shared features between *P. ovale* and *P. vivax*, including enlarged host erythrocytes and Schüffner-like dots. Because Figure 2 displays a single representative cross-validation fold rather than pooled counts, the absolute error counts shown differ slightly from, though are directionally consistent with, the fold-averaged class-level metrics reported in Table 6.

ResNetViT showed competitive performance, with F1-scores of 0.965 (*P. falciparum*), 0.930 (*P. malariae*), 0.926 (*P. ovale*), and 0.949 (*P. vivax*). Particularly noteworthy is its recall on *P. malariae* (0.970) and *P. vivax* (0.978), which were the highest among all three models for these species, indicating that the hybrid architecture is especially sensitive to the morphological characteristics of these species. ViT consistently underperformed relative to the other two architectures across all classes, with F1-scores of 0.945 (*P. falciparum*), 0.900 (*P. malariae*), 0.891 (*P. ovale*), and 0.928 (*P. vivax*).

**Figure 3:**
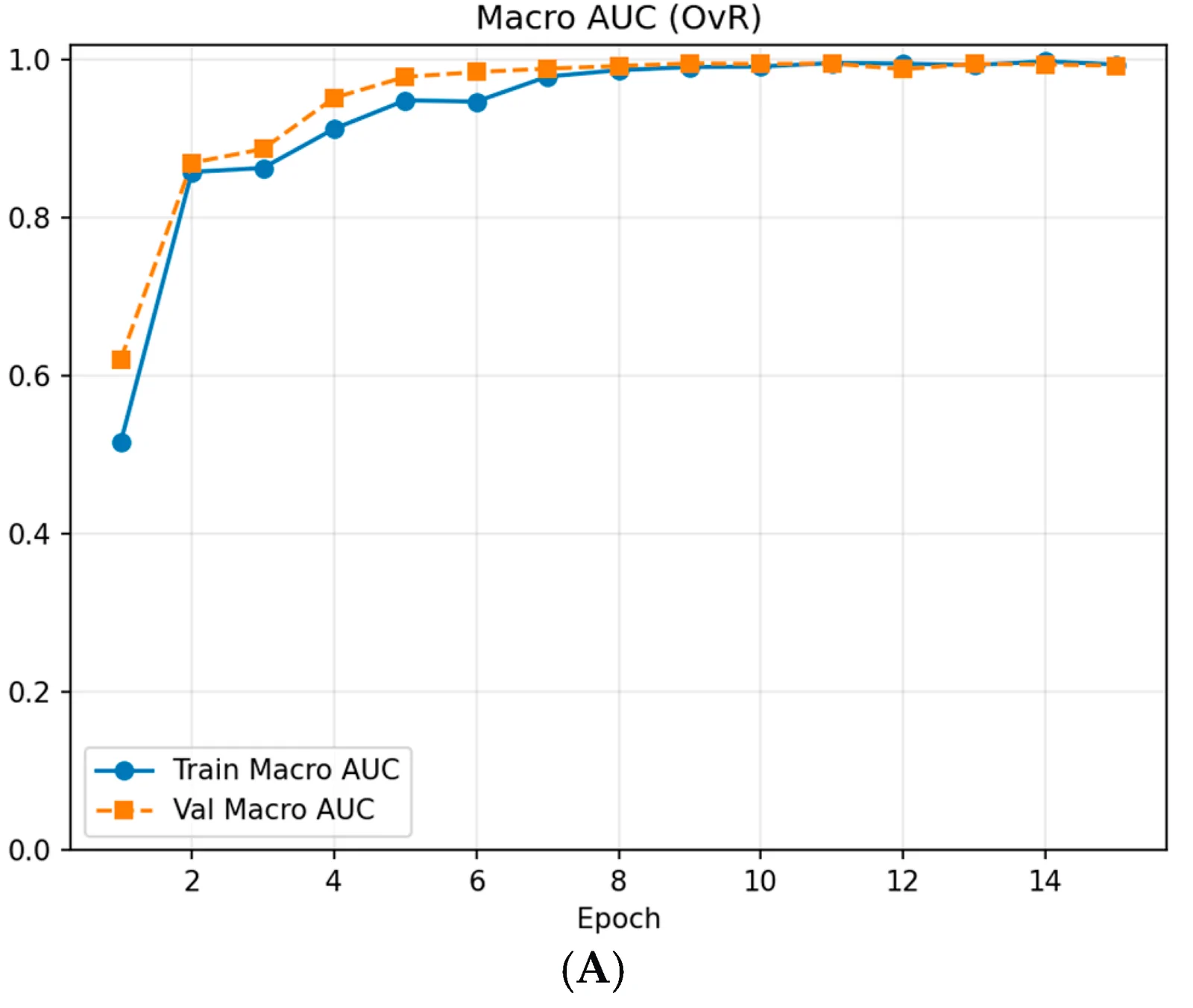

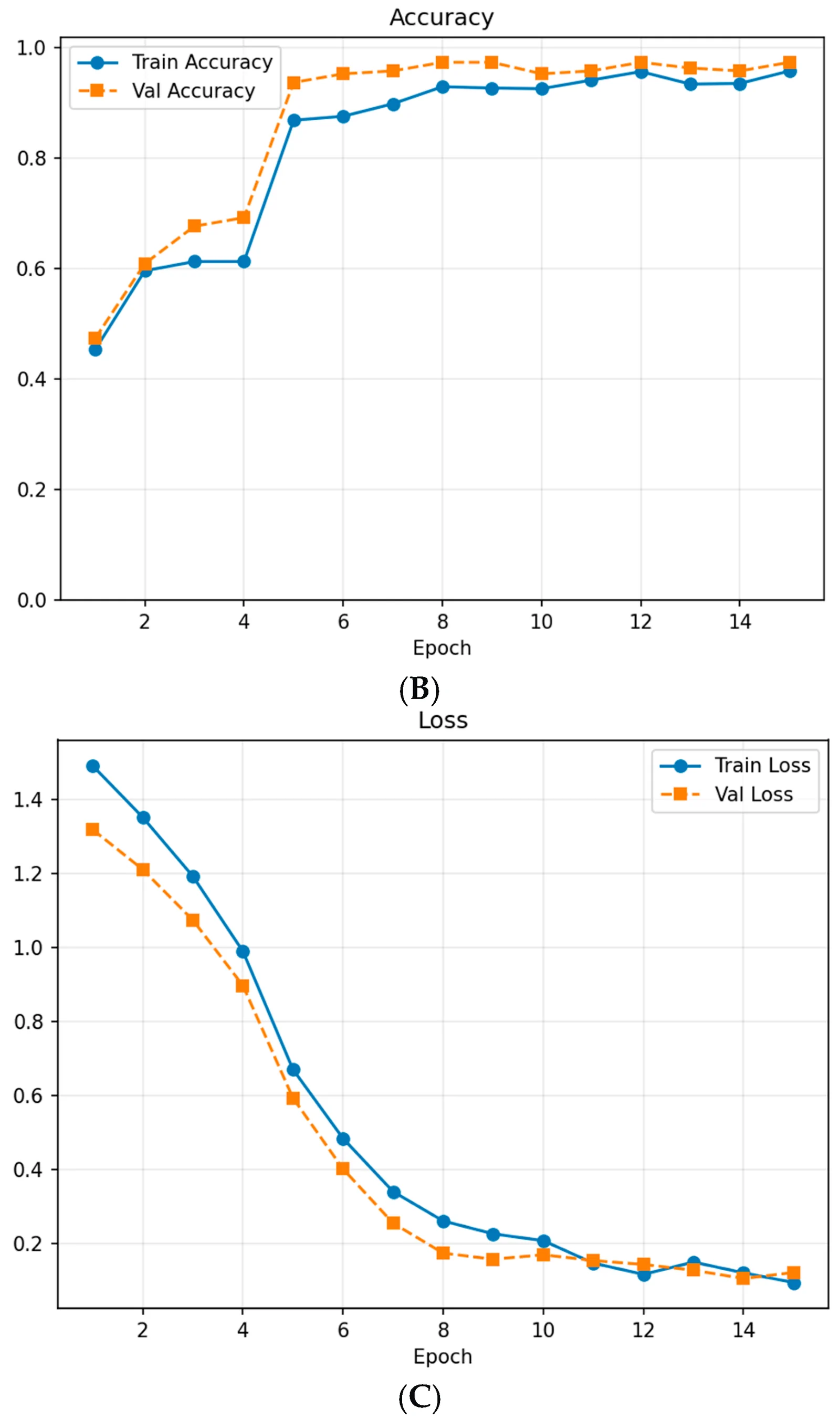
Training dynamics of the ResNet50 model over 14 epochs. (**A**) Macro-averaged ROC-AUC curves. Validation AUC reaches 1.00 from epoch 8 onward, indicating excellent discriminative ability. (**B**) Accuracy curves. Validation accuracy stabilises at 0.97 from epoch 8 onward. (**C**) Loss curves. Both training and validation loss decrease steadily and remain closely aligned, demonstrating stable optimisation and good generalisation without overfitting.

The training dynamics of the ResNet50 model are illustrated in Figure 3, which show macro-averaged ROC-AUC, accuracy, and loss curves for both training and validation sets over 14 epochs (early stopping after epoch 14). The ROC-AUC curves (Figure 3A) show a rapid increase: training AUC rises from 0.52 at epoch 1 to 0.96 by epoch 5, reaching 1.00 from epoch 9 onward; validation AUC follows closely, increasing from 0.63 to 0.98 by epoch 5 and achieving 1.00 from epoch 8 onward. The accuracy curves (Figure 3B) demonstrate that training accuracy improves from 0.45 to 0.88 by epoch 5, stabilising around 0.94–0.95 thereafter, while validation accuracy increases from 0.46 to 0.92 by epoch 5 and reaches 0.97 from epoch 8 onward. The loss curves (Figure 3C) show a sharp decrease during the first five epochs, training loss from 1.50 to 0.70, validation loss from 1.30 to 0.60, followed by a steady decline to 0.09 and 0.10 by epoch 14. The close alignment between training and validation curves across all three metrics confirms that ResNet50 generalises well without overfitting.

**Figure 4:**
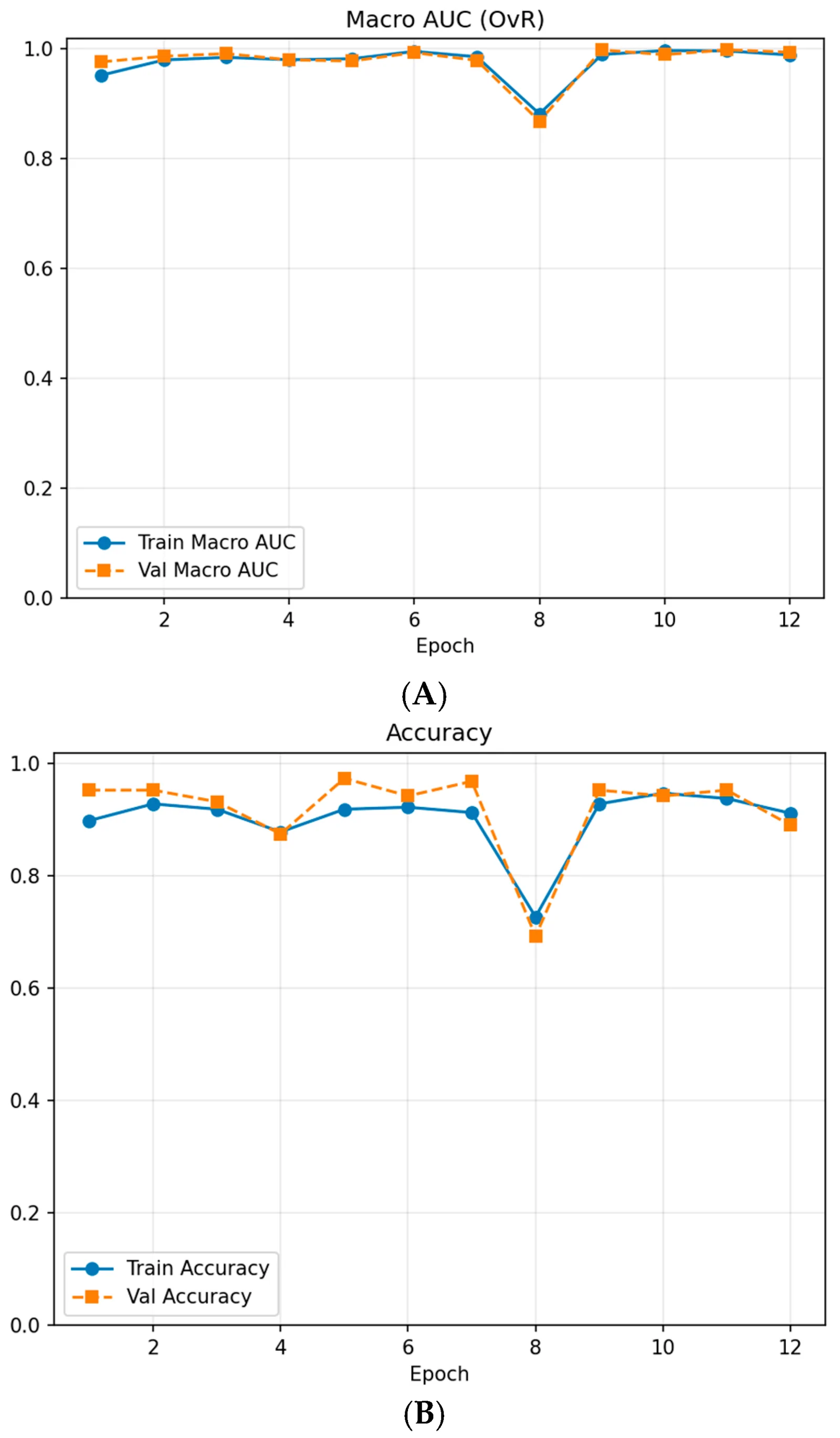

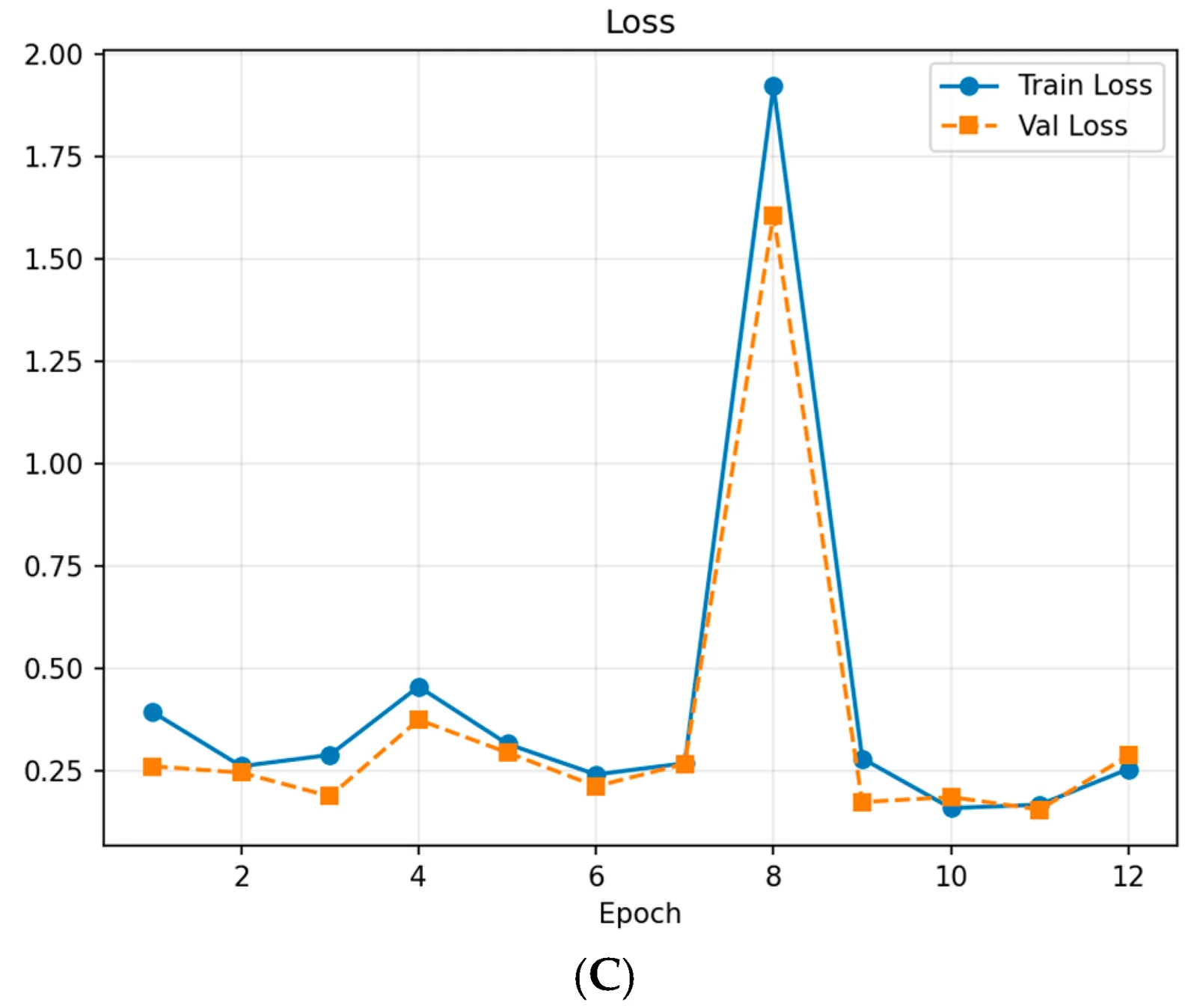
Training dynamics of the ViT model over 12 epochs. (**A**) Macro-averaged ROC-AUC curves. Validation AUC shows notable instability, dropping to 0.88 at epoch 8 before recovering to 0.99. (**B**) Accuracy curves. Validation accuracy exhibits a sharp drop at epoch 8 (from 0.97 to 0.72), indicating optimisation instability. (**C**) Loss curves. Validation loss spikes at epoch 4 and again at epoch 8, confirming the sensitivity of the transformer architecture to the small-data microscopy regime.

Figure 4 presents the training dynamics of the standalone ViT model, displaying macro-averaged ROC-AUC, accuracy, and loss curves for both training and validation sets over 12 epochs, with early stopping applied after epoch 12. The ROC-AUC curves (Figure 4A) show that both training and validation AUC reach high values quickly, with training AUC at 0.94 and validation AUC at 0.96 by epoch 1. Training AUC remains consistently high, fluctuating between 0.96 and 0.99 throughout training. However, validation AUC exhibits notable instability: after reaching 0.99 at epoch 6, it drops sharply to 0.88 at epoch 8 before recovering to 0.99 by epoch 11. These fluctuations indicate that the transformer architecture, despite achieving strong final performance, exhibits less stable convergence compared to the convolutional baseline.

The accuracy curves (Figure 4B) reveal a concerning pattern. Training accuracy remains consistently high, ranging between 0.90 and 0.96 after the first epoch. Validation accuracy, however, shows a dramatic drop at epoch 8, falling from 0.97 to 0.72, followed by a recovery to 0.97 by epoch 9. This sudden degradation and subsequent recovery suggest that the ViT model is sensitive to specific batches or features during training, potentially due to the limited size of the microscopy dataset. The temporary gap between training and validation accuracy at epoch 8 (0.96 vs. 0.72) is a clear sign of optimisation instability or transient overfitting.

The loss curves (Figure 4C) confirm these observations. Both training and validation loss decrease rapidly during the first three epochs, with validation loss reaching 0.20 by epoch 3. However, at epoch 4, validation loss spikes to 0.35 while training loss increases to 0.45, indicating a temporary divergence. After epoch 8, both losses stabilise at low values (0.25–0.30). These instabilities, entirely absent in ResNet50, indicate that the transformer architecture is more sensitive to the small-data regime and may require larger datasets, stronger regularisation, or more careful learning rate scheduling to achieve stable convergence. Despite these fluctuations, the ViT model ultimately achieves acceptable performance, though with lower aggregate metrics than both ResNet50 and the hybrid ResNetViT.

**Figure 5:**
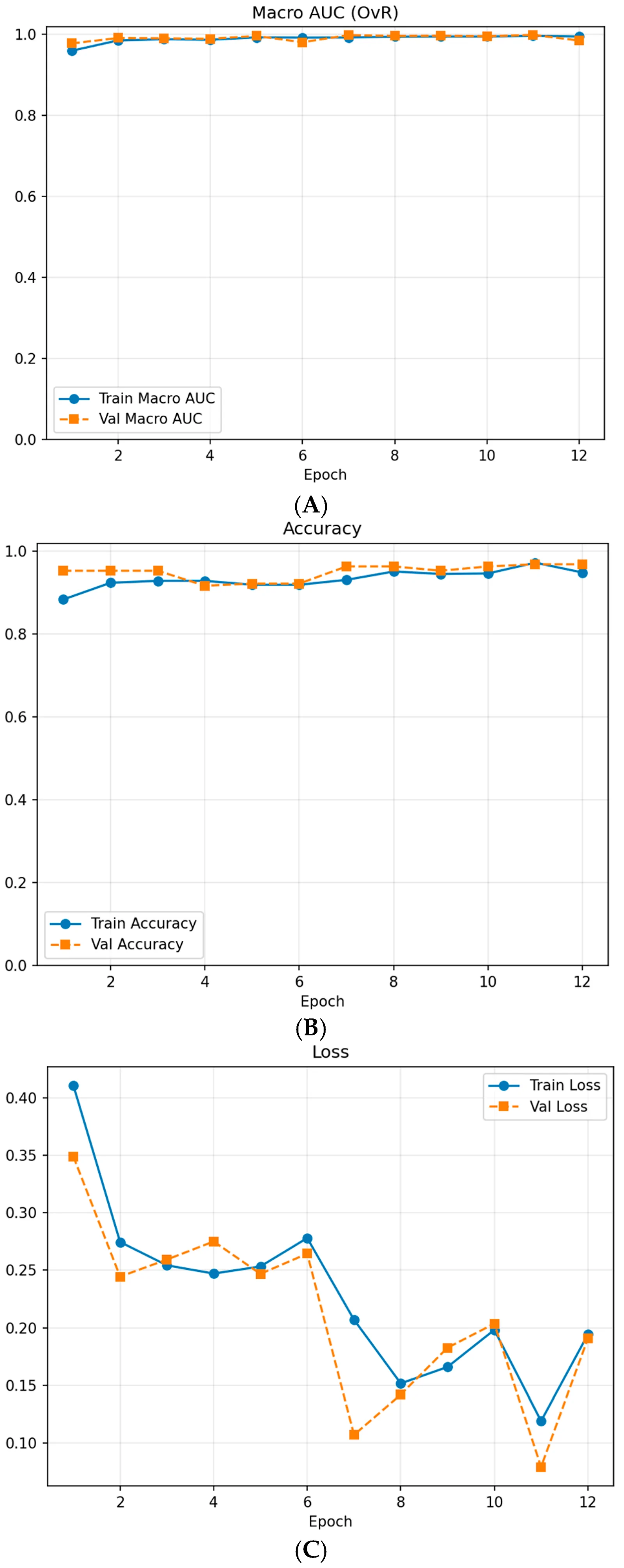
Training dynamics of the hybrid ResNetViT model over 12 epochs. (**A**) Macro-averaged ROC-AUC curves. Validation AUC reaches 1.00 from epoch 3 onward, with no fluctuations. (**B**) Accuracy curves. Validation accuracy remains stable at 0.96–0.97 throughout training, with no drops or instability. (**C**) Loss curves. Both training and validation loss decrease steadily without spikes, demonstrating stable optimisation and strong generalisation. The hybrid architecture combines the stability of ResNet50 with the representational power of ViT.

Figure 5 illustrates the training dynamics of the hybrid ResNetViT model, reporting macro-averaged ROC-AUC, accuracy, and loss curves for both training and validation sets over 12 epochs (early stopping after epoch 12). The ROC-AUC curves (Figure 5A) demonstrate rapid and stable convergence. Training AUC reaches 0.96 by epoch 1 and 0.99 by epoch 3, achieving 1.00 from epoch 5 onward. Validation AUC follows even more closely, reaching 0.98 by epoch 1, 1.00 by epoch 3, and maintaining perfect discriminative ability thereafter. Unlike the ViT model, the hybrid architecture shows no fluctuations or drops in validation AUC, indicating that the combination of local convolutional features with global transformer representations stabilises training.

The accuracy curves (Figure 5B) show consistent and stable improvement. Training accuracy rises from 0.88 at epoch 1 to 0.94 by epoch 2, stabilising at 0.94–0.96 for the remainder of training. Validation accuracy starts at 0.96 from epoch 1 and remains stable at 0.96–0.97 throughout all 12 epochs, with no drops or fluctuations. This remarkable stability contrasts sharply with the ViT model, which exhibited a dramatic drop in validation accuracy at epoch 8. The hybrid architecture’s ability to maintain high validation accuracy from the very first epoch suggests that the fused feature representation provides a strong inductive bias that generalises well even with limited data.

The loss curves (Figure 5C) confirm the stable optimisation of the hybrid model. Both training and validation loss decrease rapidly during the first three epochs, with training loss falling from 0.41 to 0.26 and validation loss from 0.35 to 0.26. Thereafter, both losses continue to decline steadily, reaching 0.12 and 0.08 by epoch 11, respectively. Notably, no spikes or divergences are observed in either curve, in contrast to the ViT model’s loss spikes at epochs 4 and 8. The close alignment between training and validation loss throughout training confirms that the hybrid architecture generalises well without overfitting, combining the stability of the convolutional backbone with the representational power of the transformer.

A comprehensive efficiency comparison across architectures is presented in Table 7, which reports computational complexity (FLOPs), total number of parameters, and Expected Calibration Error (ECE). ResNet50 is the most efficient model, with 15.27 G FLOPs and 1.49 million parameters, but shows the highest ECE (0.0577), indicating moderate miscalibration. ViT is substantially larger, with 1319.56 G FLOPs and 88.80 million parameters, achieving a lower ECE of 0.0464. The hybrid ResNetViT has the highest computational footprint (1334.83 G FLOPs) and parameter count (91.53 million) but achieves the best calibration, with an ECE of 0.0437.

**Figure 6:**
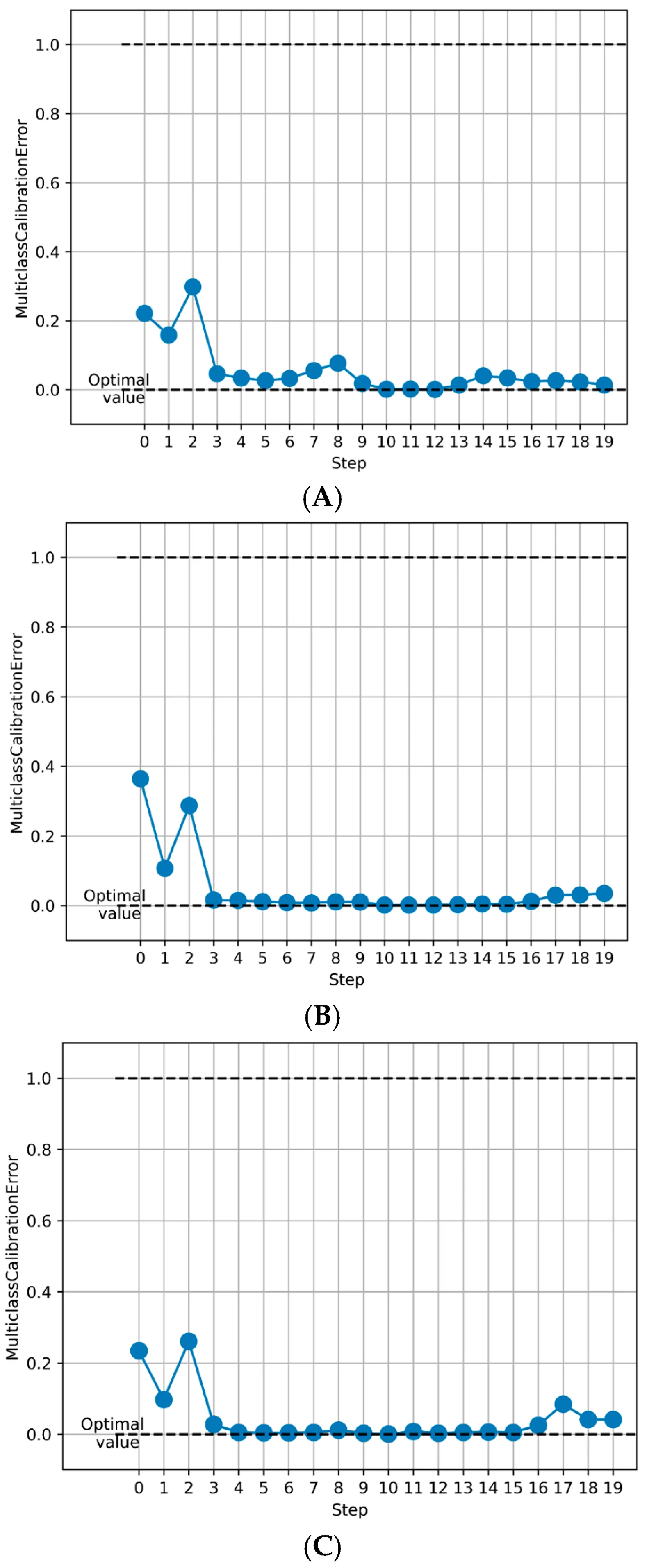
Multiclass calibration error as a function of training steps for ResNet50 (**A**), ViT (**B**), and ResNetViT (**C**). The dashed horizontal line indicates the ideal calibration error of zero (perfect calibration), serving as a visual reference for convergence. ResNet50 shows a brief warmup phase with moderate fluctuations before stabilising around 0.01–0.02. ViT exhibits the highest initial error (0.37) and a pronounced spike at step 2 (0.30), reflecting training instability typical of transformer architectures on small datasets. The hybrid ResNetViT converges smoothly and rapidly without any spikes, maintaining steady error during the first three steps before reaching minimal calibration by step 4. These dynamics demonstrate the superior calibration stability of the hybrid architecture, making it the most reliable choice for clinical applications.

Figure 6 illustrates the multiclass calibration error as a function of training steps for ResNet50, ViT, and the hybrid ResNetViT, respectively.

ResNet50 (Figure 6A) exhibits a calibration path characterised by an initial warmup phase followed by rapid stabilisation. The error starts at 0.22 at step 0, decreases slightly to 0.17 after the first step, then briefly rises to a peak of 0.33 at step 2. This initial fluctuation suggests that the model is exploring the parameter space and adjusting its confidence estimates during the earliest phase of training. From step 3 onward, the error drops sharply to 0.08 and continues to decline steadily, reaching 0.02 by step 6 and stabilising around 0.01–0.02 from step 9 to the end of training. This pattern indicates that once ResNet50 overcomes the initial warmup period, it converges quickly to a well-calibrated state and maintains it consistently.

ViT (Figure 6B) displays a markedly different and less stable calibration trajectory. The initial error is considerably higher, starting at 0.37 at step 0—nearly 70% higher than ResNet50’s starting point. This elevated initial error reflects the transformer architecture’s lack of inherent inductive biases for image data, requiring more learning to align confidence with accuracy. The error drops to 0.12 at step 1, suggesting rapid initial learning, but then spikes sharply to 0.30 at step 2. This spike coincides with the accuracy drop and loss increase observed in Figure 4A–C, confirming a transient period of instability. A second, smaller elevation appears at step 3 (0.26) before the error finally collapses to 0.02 at step 4. From step 5 onward, the error remains stable around 0.01–0.02. The ViT model thus undergoes a volatile calibration phase during the first four steps—characterised by high initial error, a pronounced spike, and delayed stabilisation—before eventually converging to good calibration. This behaviour highlights the challenges of training transformer models on small microscopy datasets, where the lack of convolutional priors leads to greater initial uncertainty and intermittent instability.

ResNetViT (Figure 6C) demonstrates the smoothest and most stable calibration dynamics among the three architectures. The error starts at 0.23 at step 0, lower than ViT and comparable to ResNet50, and remains remarkably steady at 0.24 through steps 1 and 2, showing none of the spikes or fluctuations seen in the other two models. At step 3, the error begins its decline, dropping to 0.02 by step 4 and stabilising at 0.01–0.02 from step 9 onward. What distinguishes the hybrid model is the absence of any abrupt changes or instabilities. Unlike ResNet50, it does not experience a midstep spike (the rise to 0.33 at step 2). Unlike ViT, it shows no elevated initial error (0.37) nor the sharp spike at step 2 (0.30). Instead, the hybrid model maintains a steady calibration error during the first three steps, then transitions smoothly and rapidly to minimal error by step 4. This behaviour reflects the complementary strengths of the two backbones: the convolutional branch provides stable local feature extraction and inductive biases that anchor the model’s confidence from the outset, while the transformer branch contributes global context without destabilising training.

### 4.2. Explaining Model Predictions

To interpret and visualise the decision-making process of the three architectures, we used the pytorch-grad-cam library (version 1.4.8). This toolkit identifies the most influential input regions contributing to model predictions by generating Class Activation Maps (CAMs). Multiple visualisation methods were applied, including GradCAM, GradCAM++, XGradCAM, GradCAMElementWise, and LayerCAM, each offering a slightly different perspective on feature importance and spatial localisation. Together, these explainers highlight which image areas guided each classifier’s output.

**Figure 7:**
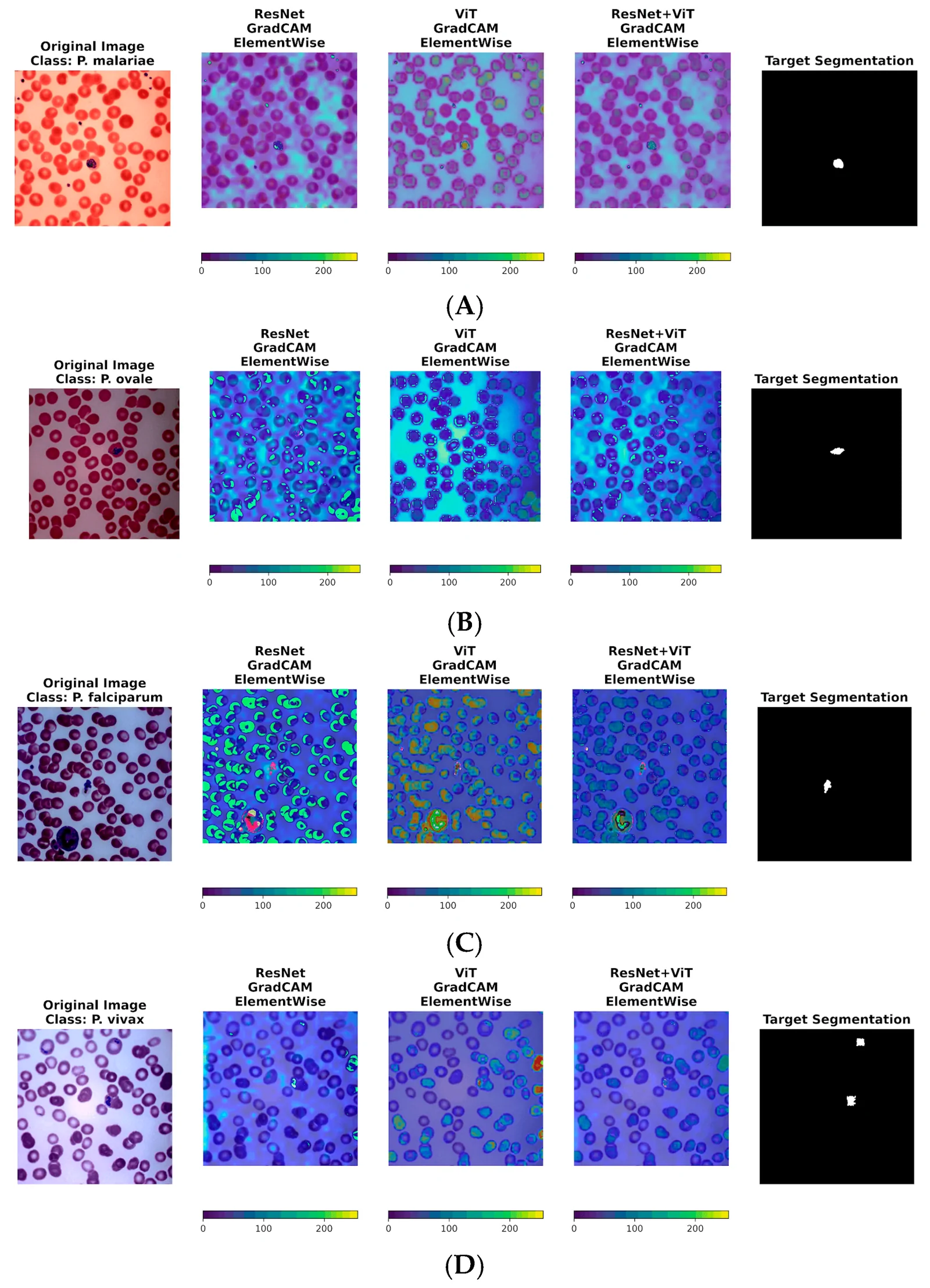
GradCAMElementWise attention maps for representative samples of four *Plasmodium* species. Each row corresponds to a species: (**A**) *P. malariae*, (**B**) *P. ovale*, (**C**) *P. falciparum*, and (**D**) *P. vivax*. For each species, five columns are shown: the original microscopy image, the attention map produced by ResNet50, the attention map produced by ViT, the attention map produced by the hybrid ResNetViT, and the ground-truth target segmentation indicating the parasite location.

Visual inspection of the CAMs reveals distinct attention patterns across the three architectures. Figure 7 illustrate the GradCAMElementWise maps for representative samples of *P. malariae*, *P. ovale*, *P. falciparum*, and *P. vivax*, respectively.

Figure 7A presents the attention maps for a representative *P. malariae* sample. The original image shows a thick smear field containing several erythrocytes, with the parasitised cell located centrally. The ResNet50 attention map activates broadly across multiple cells and background regions, including uninfected erythrocytes and debris. The ViT attention map is more compact and centred on the infected erythrocyte. The hybrid ResNetViT attention map appears qualitatively more consistent with the ground-truth annotation, concentrating on the parasitised cell. Figure 7B shows attention maps for a *P. ovale* sample. The original image contains a parasitised erythrocyte with an enlarged, oval shape, and Schüffner-like dots visible in the cytoplasm. ResNet50 produces diffuse activation that extends beyond the infected cell into adjacent areas, including a neighbouring uninfected erythrocyte. ViT focuses on the infected cell. The hybrid ResNetViT attention map appears qualitatively more consistent with the ground-truth annotation, highlighting the infected erythrocyte. Figure 7C shows attention maps for a *P. falciparum* sample. The original image displays multiple ring forms within a single erythrocyte, with some parasites located at the erythrocyte periphery. ResNet50 activates across a wide area, including uninfected cells and background regions. ViT produces tighter attention centred on the infected erythrocyte. The hybrid ResNetViT attention map appears qualitatively more consistent with the ground-truth annotation, capturing the ring forms within the infected cell. Figure 7D shows attention maps for a *P. vivax* sample. The original image displays an enlarged erythrocyte containing a large, amoeboid trophozoite with irregular cytoplasmic extensions, along with Schüffner’s dots visible throughout the host cell cytoplasm. ResNet50 produces widespread activation extending beyond the infected cell. ViT produces attention centred on the infected erythrocyte. The hybrid ResNetViT attention map appears qualitatively more consistent with the ground-truth annotation, highlighting the infected cell and its internal structures.

**Figure 8:**
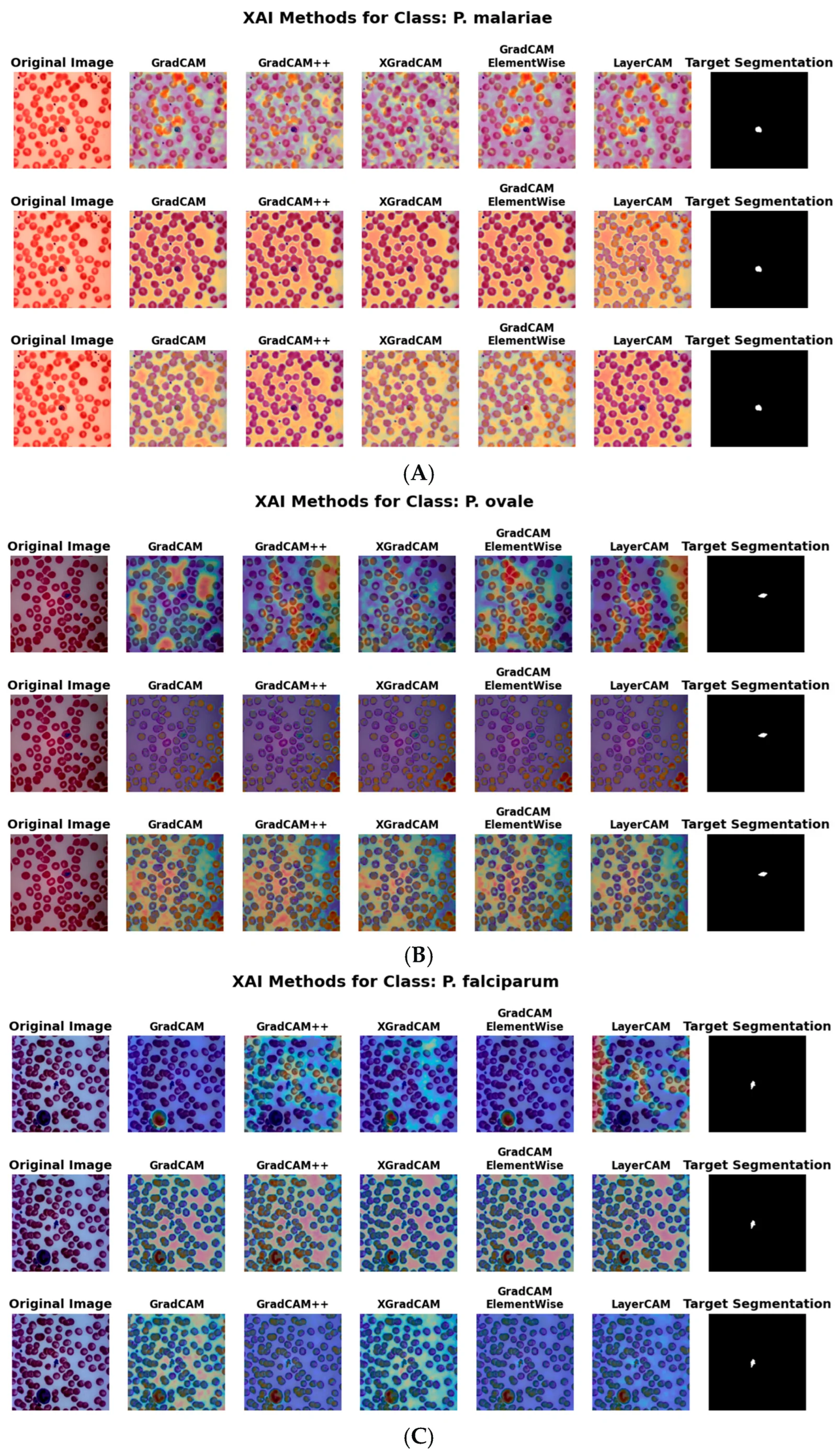

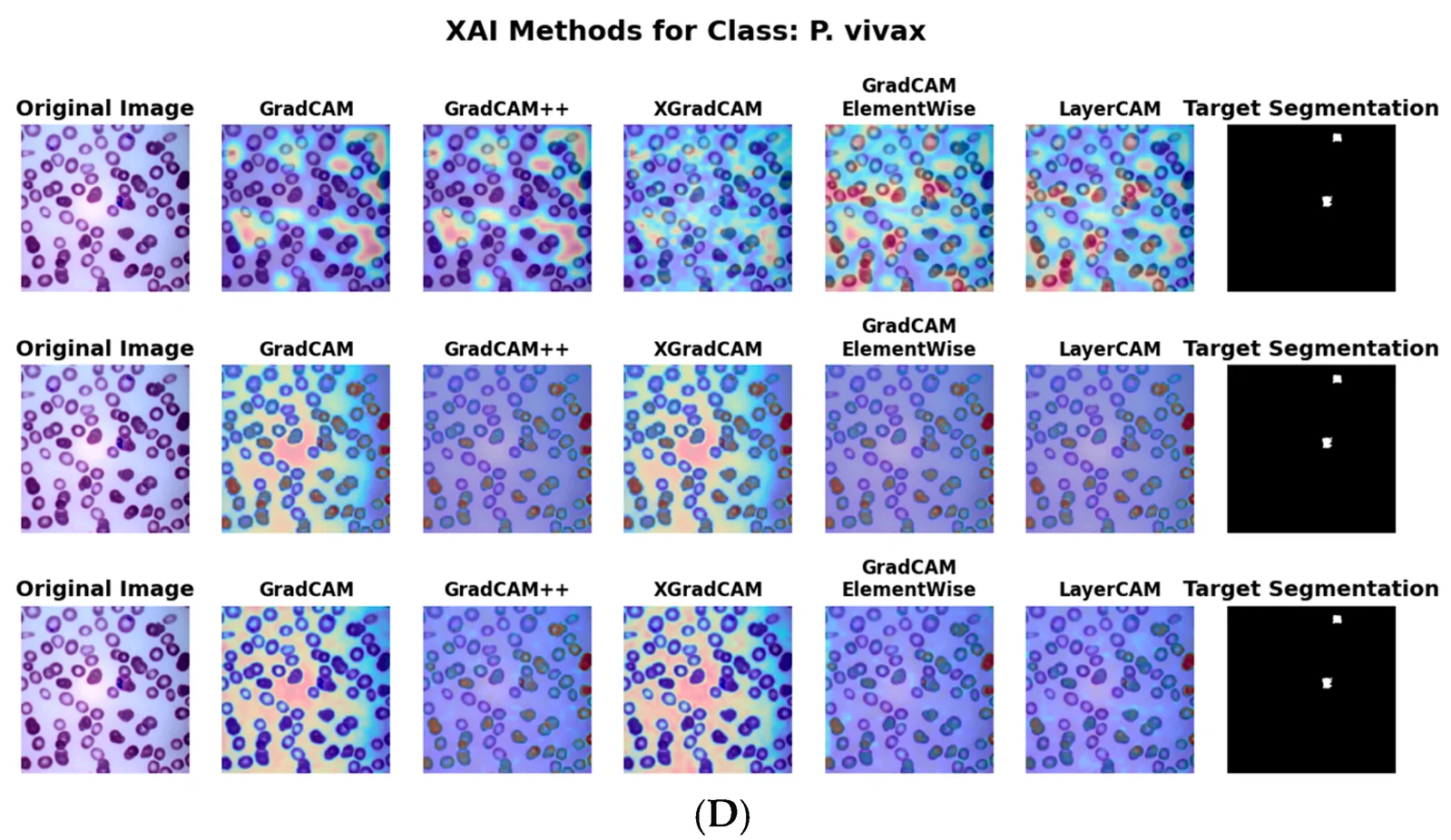
Comparison of five XAI methods across four *Plasmodium* species. Rows: ResNet50 (**top**), ViT (**middle**), hybrid ResNetViT (**bottom**). Columns: original image, five CAM methods, and ground truth. Each subfigure corresponds to a different species: (**A**) *P. malariae*, (**B**) *P. ovale*, (**C**) *P. falciparum*, and (**D**) *P. vivax*. Based on qualitative visual inspection, the hybrid model tends to produce attention maps that are more consistent with parasite locations than either single backbone, regardless of the XAI method used; this comparison is qualitative, since only centroid annotations (not full segmentation masks) are available. Colour scale ranges from blue (low attention) to red (high attention), indicating which regions most influenced the model’s prediction.

A broader comparison of different CAM-based explainers is presented in Figure 8 for representative samples of the four *Plasmodium* species. Figure 8A presents the comparison for a *P. malariae* sample. For ResNet50 (first row), all five methods produce broadly similar activation patterns, with GradCAM and LayerCAM showing slightly more diffuse activation compared to the sharper localisation of GradCAMElementWise. For ViT (second row), the different methods yield more variable maps: XGradCAM and LayerCAM produce more compact attention than standard GradCAM, while GradCAMElementWise provides a balanced localisation. For the hybrid ResNetViT (third row), all methods converge to a broadly consistent pattern that, on visual inspection, appears more consistent with the target annotation. Figure 8B shows the comparison for a *P. ovale* sample. ResNet50 (first row) exhibits diffuse activation across all methods, with activation spilling into adjacent uninfected cells and background areas. ViT (second row) produces more focused attention, though GradCAM and GradCAM++ show some variability in the precise localisation of the parasite. The hybrid ResNetViT (third row) shows the most consistent visual agreement with the target annotation across all five methods, with LayerCAM and GradCAMElementWise producing particularly sharp maps. Figure 8C presents the comparison for a *P. falciparum* sample. ResNet50 (first row) activates broadly across all methods, often highlighting multiple ring forms but also including surrounding debris. ViT (second row) produces tighter attention, though the maps vary by method: GradCAM and XGradCAM tend to highlight only a subset of ring forms, while GradCAMElementWise and LayerCAM capture a broader extent of parasite structures. The hybrid ResNetViT (third row) shows visual agreement with the target annotation across all methods, capturing multiple ring forms including those at the erythrocyte periphery. Figure 8D shows the comparison for a *P. vivax* sample. ResNet50 (first row) produces widespread activation extending well beyond the infected cell for all five methods. ViT (second row) generates more compact, cell-centred attention, though some methods (particularly GradCAM) produce activation that is narrower in scope, missing portions of the amoeboid trophozoite. The hybrid ResNetViT (third row) shows visual agreement with the target annotation across all methods, with GradCAMElementWise and LayerCAM producing maps that capture the full irregular morphology of the *P. vivax* trophozoite, including its cytoplasmic extensions.

It is important to note that the available ground truth consists of centroid annotations of parasites rather than full pixel-level segmentations. Consequently, CAM ground-truth alignment can only be assessed visually, and overlap-based metrics such as the Dice score cannot be reliably computed. This limitation means that the current analysis may underestimate or misrepresent the true extent of model attention. A more quantitative and clinically meaningful evaluation of explainability would require fully segmented masks or multiexpert morphological annotations. By comparing the different explainers, these results, while inherently qualitative, confirm the previous findings.

## 5. Discussion

This study systematically evaluated three deep learning architectures, a convolutional baseline (ResNet50), a ViT, and a hybrid ResNetViT, for *Plasmodium* species classification on real-world thick blood smear images combined with publicly available data. All models achieved consistently high performance across cross-validated metrics, underscoring the strong potential of deep learning for automated malaria diagnosis in a challenging, multiclass, thick-smear setting. Although the observed performance is slightly lower than that reported in recent binary or single-cell studies, this discrepancy is expected given the increased task complexity and the use of non-segmented, heterogeneous thick-smear patches rather than curated single-cell crops. The comparative analysis revealed distinct yet complementary strengths across the evaluated architectures. ResNet50 exhibited remarkable stability and robustness, achieving consistently high precision and accuracy across cross-validation folds. Its training dynamics showed smooth convergence with steadily decreasing loss and closely aligned training/validation curves, confirming strong generalisation without overfitting. ViT, despite achieving high accuracy and F1-score, exhibited notable training instability, with sharp drops in validation accuracy and corresponding spikes in validation loss at specific epochs. These fluctuations indicate that the transformer architecture is more sensitive to the small-data regime and may require larger datasets or stronger regularisation to achieve stable convergence. The hybrid ResNetViT achieved the best calibration profile and the most stable training dynamics, combining the stability of the convolutional backbone with the representational power of the transformer. Its validation metrics reached optimal values rapidly and remained stable throughout training, with no fluctuations or drops.

To better contextualise the clinical relevance of model errors, class-level precision, recall, and F1-scores were analysed together with confusion matrices. This analysis is particularly important because not all species-level errors have equivalent clinical implications. Misclassification of *P. falciparum* as a non-*falciparum* species may be especially consequential due to its association with severe disease and the need for prompt treatment. ResNet50 achieved very high precision for *P. falciparum* and very high recall, with only a small number of *P. falciparum* samples misclassified as *P. vivax* or *P. ovale*. Notably, no *P. vivax* samples were misclassified as *P. falciparum*, indicating an asymmetric error pattern that favours clinical safety by minimising false negatives for the most dangerous species. The hybrid model showed superior recall on *P. malariae* and *P. vivax*, the highest among all three models for these species, suggesting that the hybrid architecture is especially sensitive to the morphological characteristics of non-*falciparum* species.

In line with the hypothesis on task specific feature learning, all architectures were trained from scratch rather than finetuned from pretrained weights. The ablation study confirmed that frozen pretrained backbones led to a marked performance drop compared with full retraining. For the convolutional and hybrid models, the drop was particularly severe, demonstrating that largescale natural image datasets have fundamentally different visual statistics from microscopic malaria images, and that domain-specific feature learning is essential. Merging proprietary and open-access datasets enhanced robustness and domain transferability. The combination of the institutional collection with a publicly available Kaggle dataset increased sample diversity, while preprocessing steps such as patch extraction, normalisation, and augmentation reduced variability due to staining and acquisition protocols. These findings emphasise that strategic data integration and normalisation are indispensable for deploying AI models under real-world clinical variability.

Better calibrated probability estimates, as observed for the hybrid architecture, are especially relevant in clinical practice, where confidence scores may guide downstream decisions such as species-specific treatment thresholds or the need for confirmatory testing. The calibration error dynamics revealed that the hybrid model converged smoothly and rapidly to minimal calibration error without the spikes observed in the standalone transformer or the fluctuations seen in the convolutional baseline. This stable calibration trajectory makes the hybrid model the most reliable choice for clinical applications where well-calibrated probability estimates are essential for safe decision-making.

The hybrid ResNetViT effectively integrates ResNet’s local feature extraction with ViT’s global context modelling. Explainability analyses revealed that ResNet50 frequently assigned substantial activation to regions outside the infected cell, suggesting reliance on contextual cues from the surrounding smear rather than exclusively on parasite morphology. ViT produced more compact, cell-centred attention, but sometimes focused too narrowly, missing portions of the parasite’s morphological features. The hybrid ResNetViT achieved the best visual alignment with ground-truth segmentations across all four species and across all XAI methods tested. This advantage illustrates the unique capability of the hybrid approach to exploit both detailed cell morphology and spatial context, significantly improving interpretability and robustness compared to single model architectures. However, this analysis remains qualitative: the available ground truth consists of parasite centroids rather than full pixel-level segmentations, preventing rigorous quantification of attention accuracy. As a result, CAMs should be interpreted as tools to assess plausibility of model attention rather than definitive localisation metrics.

The present results compare favourably with the existing literature. While many previous studies focused on binary infected/uninfected classification or species identification on thin smears using curated single-cell crops, the current work addresses the more challenging task of species-level classification on real, non-segmented thick smears. The hybrid architecture’s performance is competitive with or superior to prior hybrid approaches evaluated on curated datasets, and the systematic calibration and explainability analyses go beyond what is typically reported. The use of slide-level grouped cross-validation to prevent data leakage addresses a common methodological shortcoming identified in recent systematic reviews.

Several limitations should be considered when interpreting the reported performance and the extent to which these findings support claims of generalisation or clinical readiness. First, the effective statistical unit in this study is the individual microscopy slide, even though most of the learning signal comes from patchified crops. With a limited number of high-resolution slides and many derived patches, the dataset is exposed to intra-slide correlation. Although Stratified Group K-Fold prevented leakage by keeping all patches from the same slide in a single fold, the grouping operates at the slide level rather than the patient level due to inconsistent identifiers across sources. Furthermore, statistical comparisons between models (DeLong and McNemar tests) were performed on pooled patch-level predictions. Consequently, residual within-slide correlation may have resulted in underestimated p-values by treating patches from the same slide as independent observations. Future studies should perform statistical inference after aggregating patch predictions into a single slide-level prediction, thereby aligning the unit of inference with the experimental design.

Second, species-level malaria diagnosis is inherently imbalanced, and minority classes such as *P. ovale* and *P. malariae* remain under-represented in the merged dataset.

Third, although combining two independent datasets increased image diversity and reduced the likelihood of overfitting to a single acquisition setting, the heterogeneity of the merged dataset may still introduce domain-specific shortcuts related to acquisition pipelines, including sensor characteristics, microscope optics, staining intensity, and compression artefacts. Consequently, internal cross-validation cannot replace evaluation on a truly independent cohort collected at different sites using different microscopes and staining protocols. Moreover, the present study did not include a leave-one-source-out evaluation in which models were trained on one source and tested exclusively on the other. Therefore, residual source-specific acquisition shortcuts cannot be completely excluded. Such cross-source validation would provide a stronger approximation to external validation and should be addressed in future work, particularly when larger datasets become available.

Fourth, the CAM-based explainability analysis is limited by the lack of full pixel-level segmentations, making quantitative assessment of attention accuracy impossible. Fifth, practical deployment aspects remain unassessed: the study does not report inference speed, compute budget, or device performance, and hybrid architectures may introduce additional memory and latency constraints critical in low-resource settings. Finally, although two heterogeneous data sources were used, the lack of independent external validation means the results should be viewed as proof-of-concept rather than evidence of clinical readiness.

Beyond the technical comparison of architectures, these findings carry practical implications for malaria diagnosis in low-resource, endemic settings, where access to expert microscopists is often limited. An automated species-level classifier could be deployed alongside conventional microscopy in peripheral laboratories and district hospitals as a second-reader or triage tool, flagging *P. falciparum* cases for urgent treatment and directing complex or discordant slides to reference centres for confirmatory review. When paired with digital microscopy or smartphone-based image acquisition, such models could support telemedicine workflows that extend specialist-level diagnostic capacity to remote or understaffed facilities, consistent with WHO priorities for scaling up quality-assured malaria diagnosis as part of broader elimination and eradication strategies. Realising this potential will nonetheless require the prospective, multisite validation and deployment studies discussed below, together with attention to infrastructure constraints (connectivity, computing hardware, and technician training) that are common in the highest-burden regions.

Future research should emphasise largescale, prospective validation using datasets collected across multiple geographical regions and healthcare centres to more accurately assess real-world performance. Expanding evaluation to new datasets will be essential to understand how the methodology behaves under diverse acquisition conditions. Another important direction is the extension of current models to clinically relevant tasks such as mixed infection detection, parasite staging, and parasitaemia quantification across both thick and thin smears. These capabilities would bring automated systems closer to the full spectrum of operational diagnostic needs. Beyond algorithmic improvements, future work should also address practical aspects of deployment, including systematic assessment of computational cost, inference time, and hardware requirements to determine feasibility in real clinical workflows, especially where resources are limited. Finally, enhancing clinical explainability, with model outputs that can be directly interpreted by microscopists and clinicians, represents an important step toward trustworthy and adoptable AI-assisted malaria diagnosis. The development of fully segmented ground-truth masks or multiexpert morphological annotations would enable more rigorous quantitative evaluation of CAM-based explainability methods.

## 6. Conclusions

This study examined convolutional, transformer-based, and hybrid deep learning architectures for *Plasmodium* species classification on real thick blood smear microscopy images, using a heterogeneous dataset that combines a curated institutional collection with publicly available data. Across slide-level cross-validation, all three models achieved high performance, confirming the suitability of deep learning for automated malaria microscopy in realistic, noncurated conditions.

The hybrid ResNetViT network delivered competitive discriminative performance and showed superior probabilistic calibration compared with single-backbone baselines. Its training dynamics demonstrated rapid and stable convergence without the fluctuations observed in the standalone transformer. CAM-based explainability analyses revealed that the hybrid model attends more consistently to morphologically meaningful regions than either individual backbone, achieving the best visual alignment with ground-truth parasite locations.

The use of a unified, multisource dataset highlights the feasibility of training performant models on relatively small numbers of unique slides, provided that cross-validation is carefully designed to avoid data leakage. Training from scratch proved essential, as frozen pretrained backbones led to marked performance degradation.

The present work should be regarded as a proof-of-concept for species-level malaria classification on real thick smears rather than a clinically ready triage tool. Nevertheless, the results demonstrate that hybrid architectures can successfully balance accuracy, calibration, and interpretability, offering a compelling path forward for AI-assisted malaria diagnosis in resource-limited settings.

## Figures and Tables

**Table 1:** Class distribution across data sources and the final merged dataset. Source (1) originally contained 92 high-resolution microscopy images. Each image was divided into nine nonoverlapping patches, yielding 828 patches. Source (2) (MPIDB, Kaggle) contributed 210 images. The merged dataset comprises 1038 samples across four *Plasmodium* species.

*Plasmodium* Species	Source (1) (Original Images)	Source (1) (Patches, 9×)	Source (2) (MPIDB Images)	Merged Dataset (Total Samples)
*P. falciparum*	41	369	104	473
*P. malariae*	12	108	37	145
*P. ovale*	16	144	29	173
*P. vivax*	23	207	40	247
Total	92	828	210	1038

**Table 2:** Training and architectural hyperparameters adopted for all experiments. Unless otherwise specified, the same optimisation settings were used for the ResNet50, ViT, and hybrid ResNetViT models.

**Training settings**	
Optimiser	AdamW (Fused)
Learning rate	5 × 10^−5^
Batch size	8
Gradient accumulation steps	8
Epochs	100
Warmup ratio	0.1
Early stopping	Patience = 10 epochs
Early stopping threshold	ΔROCAUC < 0.01
Loss criterion	Weighted Cross-Entropy Loss
**ViT**	
Patch size	16
Hidden size	768
Number of hidden layers	12
Number of attention heads	12
Intermediate size (MLP)	3072
**ResNet50**	
Input channels	3
Hidden sizes	[64, 128, 256, 512]
Depths	[3, 4, 6, 3]
Activation function	ReLU
Classifier dropout	0.1

**Table 3:** Model performance across five cross-validation folds (k = 5). Reported values are mean performance metrics with 95% confidence intervals in brackets. All metrics except accuracy are macro-averaged across the four *Plasmodium* species.

Metric	ResNet50	ViT	ResNetViT
Accuracy (%)	96.9 [95.6, 98.1]	93.1 [89.2, 96.7]	95.2 [91.8, 98.5]
F1-score (%)	96.3 [94.8, 97.9]	91.7 [85.7, 97.7]	94.2 [90.0, 98.8]
ROC-AUC	0.997 [0.993, 1.000]	0.989 [0.980, 0.998]	0.995 [0.991, 1.000]
Precision (%)	95.7 [91.5, 98.3]	91.7 [85.6, 97.8]	93.4 [87.5, 99.3]
Recall (%)	97.2 [96.5, 97.9]	92.8 [88.1, 97.5]	95.5 [92.6, 98.4]

**Table 4:** Pairwise comparisons of model performance using the DeLong test (for AUC differences across individual classes) and the McNemar test (for overall classification agreement). AUC values are reported per class (0: *P. falciparum*, 1: *P. malariae*, 2: *P. ovale*, 3: *P. vivax*). Raw and corrected *p*-values (Benjamini–Hochberg correction) are shown; significant differences (*p*_corr < 0.05) are highlighted in bold. McNemar test results are reported for each pairwise comparison.

Comparison	Class	AUC(A)	AUC(B)	ΔAUC	z	*p* (Raw)	*p* (Corr)	McNemar
ResNet50 vs. ViT	0	0.9978	0.9756	0.0222	−4.7245	2.3 × 10^−6^	**0.0000**	χ^2^ = 24.47, *p* = 7.5 × 10^−7^
ResNet50 vs. ViT	1	0.9970	0.9794	0.0176	−2.1500	0.0316	**0.0316**	
ResNet50 vs. ViT	2	0.9982	0.9890	0.0093	−3.7864	0.00015	**0.0003**	
ResNet50 vs. ViT	3	0.9990	0.9883	0.0107	−4.0469	5.2 × 10^−5^	**0.0002**	
ResNet50 vs. ResNetViT	0	0.9978	0.9926	0.0052	−2.3117	0.0208	0.0832	χ^2^ = 5.22, *p* = 0.0223
ResNet50 vs. ResNetViT	1	0.9970	0.9867	0.0103	−1.3739	0.1695	0.3389	
ResNet50 vs. ResNetViT	2	0.9982	0.9976	0.0007	−0.9713	0.3314	0.3389	
ResNet50 vs. ResNetViT	3	0.9990	0.9968	0.0022	−1.6417	0.1006	0.3019	
ViT vs. ResNetViT	0	0.9756	0.9926	−0.0170	3.6089	0.00031	**0.0012**	χ^2^ = 6.49, *p* = 0.0109
ViT vs. ResNetViT	1	0.9794	0.9867	−0.0073	0.8120	0.4168	0.4168	
ViT vs. ResNetViT	2	0.9890	0.9976	−0.0086	3.4914	0.00048	**0.0014**	
ViT vs. ResNetViT	3	0.9883	0.9968	−0.0085	3.3187	0.00090	**0.0018**	

**Table 5:** Ablation study comparing training strategies. Reported values are macro-averaged F1-scores with 95% confidence intervals in brackets. “Finetune Classifier” indicates that the backbone was frozen and only the classifier head was trained using ImageNet pretrained weights. “Retraining” indicates full training of all network parameters from scratch.

Model	Finetune Classifier	Retraining
ResNet50	0.663 [0.548, 0.778]	0.967 [0.937, 0.996]
ViT	0.862 [0.835, 0.889]	0.926 [0.900, 0.953]
ResNetViT	0.379 [0.208, 0.550]	0.947 [0.927, 0.967]

**Table 6:** Class-level performance metrics across models.

Class	Model	Precision	Recall	F1
0 (*P. falciparum*)	ResNet50	0.998	0.957	0.977
	ViT	0.967	0.935	0.945
	ResNetViT	0.994	0.939	0.965
1 (*P. malariae*)	ResNet50	0.935	0.976	0.954
	ViT	0.886	0.923	0.900
	ResNetViT	0.899	0.970	0.930
2 (*P. ovale*)	ResNet50	0.922	0.983	0.951
	ViT	0.874	0.934	0.891
	ResNetViT	0.923	0.932	0.926
3 (*P. vivax*)	ResNet50	0.971	0.974	0.972
	ViT	0.941	0.920	0.928
	ResNetViT	0.921	0.978	0.949

**Table 7:** Model efficiency and calibration metrics.

Model	FLOPs (G)	Params (M)	ECE
ResNet50	15.27	1.49	0.0577
ViT	1319.56	88.80	0.0464
ResNetViT	1334.83	91.53	0.0437

## Data Availability

The anonymized image dataset from Source (1) is open-source and freely available at: https://doi.org/10.5281/zenodo.18862635 (accessed on 10 February 2026). The dataset from Source (2) is publicly accessible at: https://www.kaggle.com/datasets/saife245/malaria-parasite-image-malaria-species (accessed on 20 January 2026). The code required to reproduce our findings is available at: https://github.com/AI4BIOCONE/malaria_classification (accessed on 6 March 2026).
